# Prevalence and Associated Factors of *Borrelia burgdorferi* Sensu Lato Exposure in Humans and Infection in Questing *Ixodes* Ticks in China: A Systematic Review and Meta-Analysis

**DOI:** 10.3390/microorganisms14071563

**Published:** 2026-07-17

**Authors:** Wei Wei, Pengcheng Bian, Lu An, Rui Shi, Di Jiao, Rigai Sa, Chengyu Ma, Guoshuai Li, Yaxian Du, Yuan Ma, Rui Wang

**Affiliations:** 1 College of Veterinary Medicine, Inner Mongolia Agricultural University, Hohhot 010000, China; weiweixs213149@163.com (W.W.); 13468740416@163.com (P.B.); 13694734117@163.com (L.A.); 13847325957@163.com (R.S.); 15148987036@163.com (D.J.); 13171418163@163.com (R.S.); machengyu@emails.imau.edu.cn (C.M.); lgs2765039033@163.com (G.L.); 13190607083@163.com (Y.D.); mayuan0820@126.com (Y.M.); 2 Key Laboratory of Clinical Diagnosis and Treatment of Animal Diseases, Ministry of Agriculture, National Animal Medicine Experimental Teaching Center, Hohhot 010000, China

**Keywords:** Lyme borreliosis, *Borrelia burgdorferi* sensu lato, prevalence, associated factors, meta-analysis

## Abstract

Lyme borreliosis has been reported across China, but data on *Borrelia burgdorferi* sensu lato exposure in humans and infection in questing *Ixodes* ticks remains fragmented and heterogeneous. This systematic review and meta-analysis aimed to estimate the pooled seroprevalence of *B. burgdorferi* sensu lato exposure in humans and infection prevalence in questing or unfed *Ixodes* ticks in China and to explore potential sources of heterogeneity. Six English- and Chinese-language databases were searched from inception to 10 September 2025. Eligible cross-sectional studies reported both the number examined and the number positive, with a minimum sample size of 30. Human analyses were restricted to serological evidence, whereas tick analyses included questing or unfed *Ixodes* ticks collected from vegetation or the environment. Random-effects models, subgroup analyses, and univariate random-effects meta-regression were applied. A total of 186 studies were included, of which 167 reported human data, 22 reported PCR-based data from questing *Ixodes* ticks, and 3 reported both human and tick data. The pooled human seroprevalence was 7% (95% CI: 6–8%), and the pooled PCR-confirmed infection prevalence in questing *Ixodes* ticks was 20% (95% CI: 10–32%). Substantial heterogeneity was observed in both analyses. For questing *Ixodes* ticks, infection prevalence showed marked geographical heterogeneity, with the highest pooled estimate observed in Northeastern China. In univariate meta-regression, Northern China showed a significantly lower estimate than Northeastern China, whereas sampling year, reported *Borrelia* genospecies, and *Ixodes* species were not significantly associated with pooled prevalence. Leave-one-out analyses indicated that the pooled estimates were not driven by any single study. These findings demonstrate that human exposure and environmental circulation of *B. burgdorferi* sensu lato have been documented across multiple regions of China but remain highly heterogeneous.

## 1. Introduction

Lyme borreliosis (LB) is a multisystem tick-borne infection that has expanded globally and remains an important public health concern [1]. Early disease often presents as erythema migrans at the bite site. Without treatment, infection may disseminate to the nervous system, joints, heart, and skin [2]. Clinically, LB is commonly described as early localized, early disseminated, and late disease, with outcomes ranging from isolated skin lesions to neuroborreliosis, carditis, Lyme arthritis, or acrodermatitis chronica atrophicans; fatal cases are rare [3]. Most patients respond to timely antibiotics, but delayed diagnosis can lead to long-lasting complications, and persistent post-treatment symptoms remain debated [4].

LB is caused by spirochetes belonging to the *Borrelia burgdorferi* sensu lato complex. Globally, *B. burgdorferi* sensu stricto predominates in North America, whereas *Borrelia afzelii* and *Borrelia garinii* account for most human cases in Europe; *B. garinii* is also the primary cause in Asia [5]. In China, LB was first recognized after human cases were reported in Heilongjiang Province in 1986 [6]. Multiple genospecies of the *B. burgdorferi* sensu lato complex have since been detected, and human infection has been mainly associated with *B. garinii*, *B. afzelii*, and related genospecies [7]. Genospecies may also influence clinical patterns: *B. afzelii* is often linked to skin manifestations, *B. burgdorferi* sensu stricto to arthritis, and *B. garinii* to neuroborreliosis [8].

Transmission of *B. burgdorferi* sensu lato depends on competent *Ixodes* vectors, competent reservoir hosts, and ecological conditions that maintain enzootic cycles. Key vectors include *Ixodes ricinus* in Europe, *Ixodes persulcatus* in Asia, *I. scapularis* in eastern North America, and *I. pacificus* in western North America [9]. During blood feeding, infected ticks can transmit spirochetes to susceptible reservoir hosts, and uninfected ticks can acquire infection from competent reservoirs during subsequent feeding [8,9]. In China, human exposure is mainly associated with *Ixodes* ticks, especially *I. persulcatus*, *I. sinensis*, and *I. granulatus*. Among them, *I. persulcatus* is considered a key vector and has been reported from northeastern China and some western, central, and eastern provinces [7,10].

Human risk is influenced by local tick density, infection prevalence in questing ticks, the distribution of competent reservoir hosts, vegetation and climate conditions, and the frequency of human activities in tick habitats [11]. Diagnosis is difficult because symptoms are variable and sometimes nonspecific [12].

In China, LB exposure or cases have been reported from many provinces according to study location or residence, although these reports do not necessarily identify the exact site of tick bite. Therefore, a systematic review and meta-analysis is needed to quantify human serological exposure to *B. burgdorferi* sensu lato and infection prevalence in questing *Ixodes* ticks in China, and to synthesize their spatial distribution, associated factors, and environmental hazard patterns.

## 2. Materials and Methods

### 2.1. Search Strategy and Study Selection

This systematic review and meta-analysis was conducted and reported in accordance with the Preferred Reporting Items for Systematic Reviews and Meta-Analyses (PRISMA) 2020 statement [13]. Six databases including Web of Science, PubMed, Wanfang, ScienceDirect, China National Knowledge Infrastructure (CNKI), and the VIP Chinese Science and Technology Journal Database (VIP), were searched from inception to 10 September 2025 for studies on *B. burgdorferi* sensu lato exposure or infection in humans and *Ixodes* ticks in China (Supplementary File S1). After de-duplication in EndNote X9, records were screened using predefined eligibility criteria.

Cross-sectional studies were eligible if they reported prevalence data with a sample size of at least 30 and provided both the number examined and the number positive. The minimum sample-size criterion was applied to each eligible original study or independent dataset, rather than to every within-study subgroup stratum. When an eligible study reported stratified data, such as tick species, Borrelia genospecies, or geographical strata, these strata were extracted to preserve the original data structure, but sparse strata were not treated as separate eligibility units. For human studies, eligible data were restricted to serological evidence of exposure to *B. burgdorferi* sensu lato. Studies relying solely on molecular or direct pathogen detection methods were excluded from the human meta-analysis. For the tick meta-analysis, eligible records were restricted to questing or unfed *Ixodes* ticks collected from vegetation or the environment and tested for *B. burgdorferi* sensu lato by PCR-based methods. Tick records based only on non-PCR detection methods, ticks collected from vertebrate hosts, engorged or feeding ticks, non-*Ixodes* hard ticks, and records with unclear collection source or feeding status were excluded.

Tick names were checked for taxonomic consistency before analysis. Obsolete or non-*Ixodes* names were not retained in the primary tick dataset. Duplicate datasets, reviews, case reports, laboratory-only studies, studies with sample size < 30, and studies without usable epidemiological data were excluded. Unpublished data were not included, and only English- or Chinese-language articles were considered.

### 2.2. Quality Assessment

The quality of the included studies was assessed using a scoring tool informed by the Grading of Recommendations Assessment, Development and Evaluation (GRADE) approach [14]. Studies were awarded 1 point for each of the following items: specifying the sampling year, having a sample size larger than 200, outlining a detailed sampling methodology, using a cross-sectional study design, and reporting data on at least four potential factors associated. Based on the total score (range 0–5), studies were classified as high quality (4–5 points), medium quality (2–3 points), or low quality (0–1 point).

### 2.3. Statistical Analysis

Quantitative analyses were performed in RStudio version 2024.12.0+467 using the *meta* package. To determine the most appropriate variance-stabilizing transformation, five transformations, including PRAW, PLN, PLOGIT, PAS, and PFT, were compared separately for human seroprevalence and prevalence in questing *Ixodes* ticks. Based on the comparative distributional diagnostics, the logit transformation was selected for human seroprevalence because PLOGIT showed the best relative performance among the five evaluated transformations, although the Shapiro–Wilk test remained statistically significant (W = 0.94181, *p* < 0.05). In contrast, the arcsine transformation was selected for prevalence in questing *Ixodes* ticks because PAS showed the best performance among the evaluated transformations and no statistically significant departure from normality (W = 0.94067, *p* = 0.2043).

Random-effects models were then used to estimate pooled prevalence and corresponding 95% confidence intervals. Heterogeneity was assessed using Cochran’s Q test and the I^2^ statistic. Small-study effects and potential publication bias were evaluated using funnel plots and Egger’s regression test. The trim-and-fill procedure was used as a supplementary assessment of funnel-plot asymmetry. Sensitivity analyses were performed using the leave-one-out method.

To explore heterogeneity, we conducted subgroup analyses and univariate random-effects meta-regression. For human studies, covariates included geographical region (Central, Eastern, Northeastern, Northern, Northwestern, Southwestern, and Western China), sampling period (1986–1995, 1996–2005, 2006–2015, and 2016–2025), age group (<20, 20–30, 30–40, 40–50, and >50 years), sex (men and women), season, history of tick bites, exposure group, residence (urban/rural), antibody type (immunoglobulin G (IgG), immunoglobulin M (IgM), and combined IgG + IgM), and reported or assay-attributed genospecies (*B. garinii*, *B. afzelii*, or mixed *B. garinii* + *B. afzelii*). Human serological methods included enzyme-linked immunosorbent assay (ELISA), indirect immunofluorescence assay (IFA), Western blot (WB), and combined testing strategies, including ELISA + WB, IFA + ELISA, and IFA + WB. Human exposure risk was categorized as low, moderate, or high according to the reported occupation, residence, and likelihood of contact with tick habitats. Low-risk groups included urban residents or populations with minimal contact with forested, pastoral, or tick-infested environments; moderate-risk groups included populations with occasional or indirect outdoor exposure; and high-risk groups included forestry workers, farmers, herders, field workers, or residents with frequent exposure to forested, grassland, pastoral, or tick-infested habitats.

For the PCR-confirmed tick dataset, subgroup analyses and univariate random-effects meta-regression were conducted to examine variation by geographical region, sampling period, reported *Borrelia* genospecies, and *Ixodes* species, where sufficient data were available. Geographical regions were classified as Northeastern, Northern, Eastern, Central, Northwestern, and Southwestern China. Sampling periods were grouped into 1996–2005, 2006–2015, and 2016–2025. Reported genospecies were analyzed according to the classifications provided in the original studies. For tick species and genospecies subgroup analyses, strata with limited denominators were retained only as descriptive, study-derived subgroup information when they originated from otherwise eligible datasets.

For harmonization, seasons were classified according to the calendar-based seasonal classification commonly used in China: spring from March to May, summer from June to August, autumn from September to November, and winter from December to February. Human seasonal analyses included spring, summer, autumn, and winter. When specific sampling months were reported, they were assigned to the corresponding seasonal category; when only a season was reported, the classification used in the original study was retained. Only studies that explicitly reported the season or month of sample collection were included in the seasonal analyses. For both human and tick studies, genospecies categories were extracted according to the identification and classification reported in the original studies.

## 3. Results

### 3.1. Search Results and Eligible Studies

**Figure 1:**
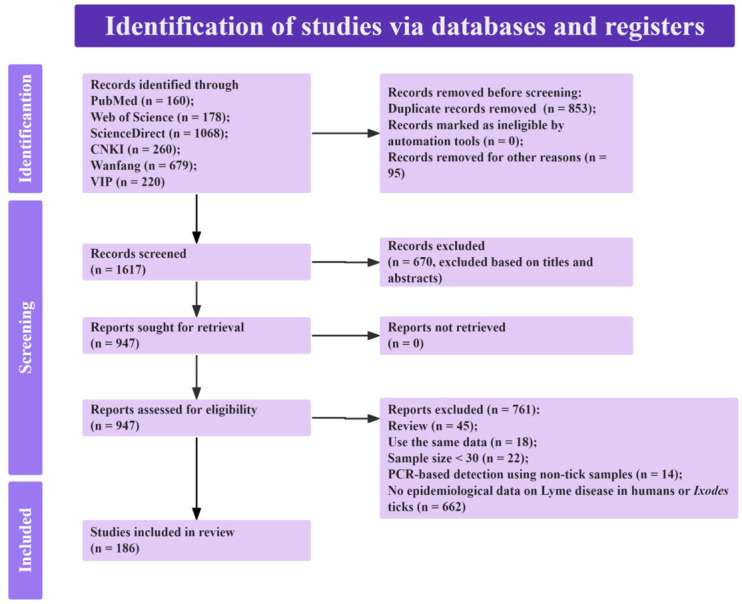
Overview of search strategy conducted for the systematic review.

A total of 2565 records were retrieved from six databases. After removing 853 duplicate records and 95 records for other reasons, 1617 records were screened by title and abstract, of which 670 were excluded. The full texts of the remaining 947 reports were assessed for eligibility. Of these, 761 reports were excluded for the following reasons: review articles (*n* = 45), duplicate datasets or use of the same data (*n* = 18), sample size < 30 (*n* = 22), tick studies using non-PCR detection methods (*n* = 14), and no usable epidemiological data on Lyme borreliosis in humans or questing *Ixodes* ticks (*n* = 662). Finally, 186 studies met the inclusion criteria and were included in the systematic review and meta-analysis (Figure 1, Supplementary File S2). Among the included studies, 167 provided human data, comprising 144,995 examined individuals and 11,867 seropositive individuals, whereas 22 provided tick data, comprising 12,862 examined ticks and 2040 PCR-positive ticks. The included studies comprised 120 high-quality, 61 medium-quality, and 5 low-quality articles. The complete reference list is provided in Supplementary File S2.

### 3.2. Publication Bias and Sensitivity Analysis

The forest plots illustrated substantial heterogeneity among both human and tick studies (Supplementary File S3). Visual inspection of the funnel plots suggested asymmetry for human seroprevalence studies and, to a lesser extent, for tick infection-prevalence studies (Supplementary File S4). The trim-and-fill procedure improved funnel-plot symmetry after imputing potentially missing studies (Supplementary File S5), suggesting that some of the observed asymmetry may have been attributable to missing small studies. Egger’s linear regression test confirmed significant funnel-plot asymmetry and small-study effects among human studies (t = −5.40, degrees of freedom (df) = 165, *p* < 0.0001; bias estimate = −4.3313, standard error (SE) = 0.8026). In contrast, no statistically significant funnel-plot asymmetry was detected among tick studies (t = 1.20, df = 20, *p* = 0.2431; bias estimate = 6.5355, SE = 5.4338; Supplementary File S6). Leave-one-out sensitivity analyses, in which individual studies were sequentially excluded, showed that the pooled prevalence estimates for both humans and ticks remained stable, indicating that no single study materially influenced the overall results and supporting the robustness of the meta-analysis findings (Supplementary Files S7 and S8).

### 3.3. Pooling and Heterogeneity Analyses

**Figure 2:**
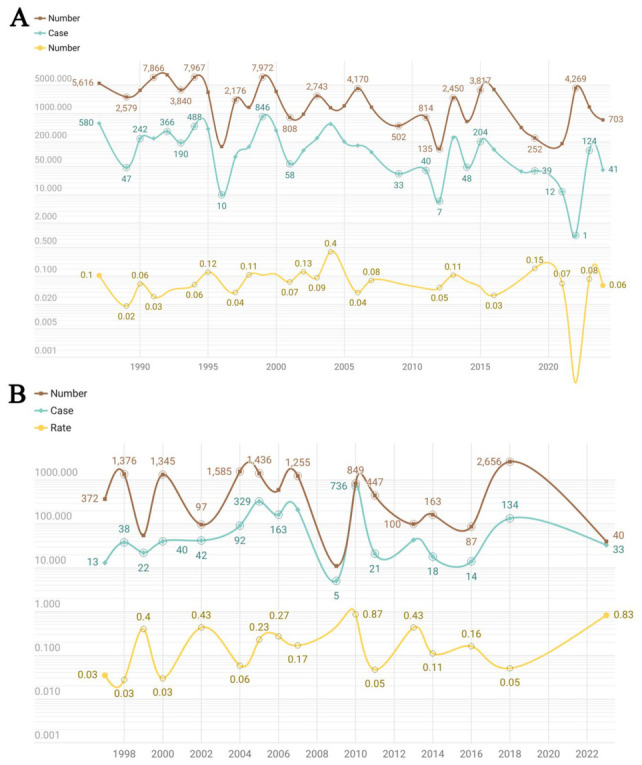
Temporal distribution of examined samples, positive samples, and prevalence of *Borrelia burgdorferi* sensu lato in humans and questing *Ixodes* ticks in China. Panel (**A**) shows annual data for humans, including examined individuals, seropositive cases, and seroprevalence. Panel (**B**) shows annual data for questing *Ixodes* ticks, including examined ticks, infected ticks, and infection prevalence. Brown lines indicate examined samples, blue-green lines indicate positive samples, and yellow lines indicate prevalence. The *y*-axis is presented on a shared logarithmic scale, with sample counts shown as absolute numbers and prevalence shown as proportions.

The annual distribution of examined samples, positive samples, and prevalence is shown in Figure 2. In humans, both the number of examined individuals and seropositive cases fluctuated across the study period, with no clear monotonic temporal trend in seroprevalence. In questing *Ixodes* ticks, the annual number of examined and infected ticks also varied markedly, and infection prevalence showed greater year-to-year variation than that observed in humans.

**Figure 3:**
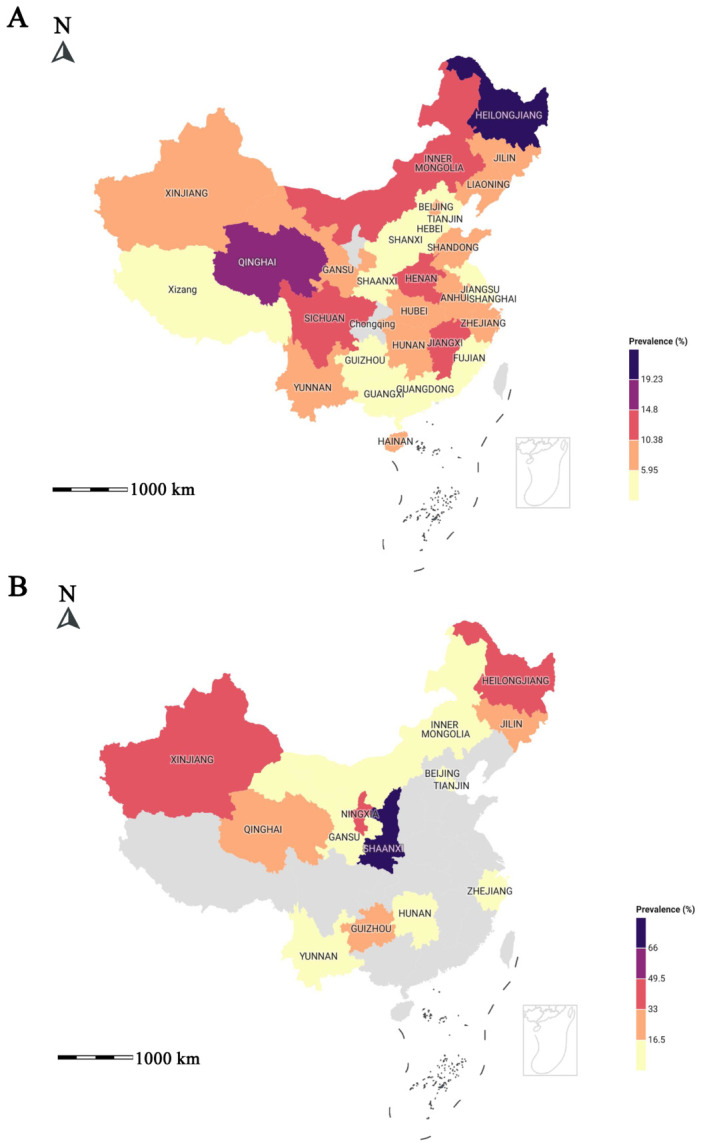
Provincial-level geographic distribution of *Borrelia burgdorferi* sensu lato prevalence in humans and questing *Ixodes* ticks in China. (**A**) Pooled seroprevalence of *B. burgdorferi* sensu lato exposure in humans across 29 provincial-level regions. (**B**) Pooled PCR-confirmed infection prevalence of *B. burgdorferi* sensu lato in questing *Ixodes* ticks across 14 provincial-level regions. The color gradient represents prevalence expressed as a percentage, with lighter colors indicating lower prevalence and darker colors indicating higher prevalence. Areas shown in gray indicate provincial-level regions for which no eligible data were available.

Provincial-level spatial distributions showed marked geographic variation in *B. burgdorferi* sensu lato prevalence in both humans and questing *Ixodes* ticks (Figure 3, Table 1). In humans, seroprevalence was reported from 29 provincial-level regions and ranged from 1.52% in Guangxi to 23.66% in Heilongjiang. Higher human seroprevalence was mainly observed in Heilongjiang, Qinghai, Sichuan, Jiangxi, Henan, Inner Mongolia, and Xinjiang, whereas lower estimates were observed in Guangxi, Shanghai, Fujian, Hebei, Tibet, and Jiangsu. In PCR-based questing *Ixodes* tick studies, infection prevalence was reported from 14 provincial-level regions and varied markedly across China, ranging from 0% in Tianjin to 82.50% in Shaanxi. The highest pooled estimate was observed in Shaanxi, followed by Ningxia, Xinjiang, Heilongjiang, Qinghai, and Jilin. Lower estimates were observed in Zhejiang, Gansu, Beijing, and Tianjin. Overall, PCR-confirmed tick infection prevalence showed greater provincial-level spatial heterogeneity than human seroprevalence.

The pooled seroprevalence of *B. burgdorferi* sensu lato exposure in humans in China was 7% (95% CI: 6–8%). Among questing *Ixodes* ticks, the pooled infection prevalence was 20% (95% CI: 10–32%). Substantial heterogeneity was observed across studies, and subgroup analyses with univariate random-effects meta-regression were conducted to explore potential sources of variation.

In humans, significant differences in seroprevalence were observed by geographical region, sampling period, age group, history of tick bites, and exposure-risk category. Geographical variation was significant in univariate meta-regression (*p* < 0.0001), with the highest pooled estimate in Northeastern China (13.10%, 95% CI: 8.38–18.70%) and lower estimates in Eastern China (5.10%, 95% CI: 3.55–6.91%) and Southwestern China (5.40%, 95% CI: 3.18–8.15%). Seroprevalence also differed by sampling period (*p* = 0.0263), with the highest estimate during 1996–2005 (9.83%, 95% CI: 7.24–12.77%) and the lowest during 2016–2025 (5.56%, 95% CI: 2.22–10.30%). Age group was significantly associated with seroprevalence (*p* = 0.0368); individuals aged 30–40 years had the highest estimate (10.19%, 95% CI: 7.72–12.96%), whereas those aged <20 years had the lowest estimate (6.73%, 95% CI: 5.06–8.62%). Participants with a reported history of tick bites had a higher seroprevalence than those without reported tick bites (10.25%, 95% CI: 8.37–12.30% vs. 5.06%, 95% CI: 2.29–8.85%; *p* = 0.0465). Exposure-risk category was also significant (*p* = 0.0466), with the highest estimate in high-risk populations (9.84%, 95% CI: 8.08–11.77%), followed by low-risk populations (8.83%, 95% CI: 4.98–13.65%) and moderate-risk populations (4.89%, 95% CI: 2.99–7.24%) (Supplementary File S9).

Other human-level covariates were not significantly associated with seroprevalence in univariate meta-regression. Seroprevalence was similar between men and women (8.58%, 95% CI: 6.97–10.33% vs. 7.97%, 95% CI: 6.32–9.79%; *p* = 0.6168). Seasonal analyses included 36 human studies, comprising 9 studies conducted in spring, 18 in summer, 7 in autumn, and 2 in winter. Seasonal estimates ranged from 5.51% in spring to 18.92% in winter, but season was not significantly associated with seroprevalence (*p* = 0.0735). The winter estimate should be interpreted cautiously because it was based on only two studies. Rural populations had a slightly higher estimate than urban populations (9.08%, 95% CI: 7.39–10.93% vs. 7.06%, 95% CI: 4.04–10.85%), although the difference was not statistically significant (*p* = 0.3758). Antibody type was not significantly associated with seroprevalence (*p* = 0.5289), with pooled estimates of 8.83% for IgG, 8.60% for IgG + IgM, and 6.61% for IgM. Reported or assay-attributed genospecies were also not significantly associated with seroprevalence (*p* = 0.7657), with pooled estimates of 10.39% for *B. garinii*, 10.19% for mixed *B. garinii* + *B. afzelii*, and 7.54% for *B. afzelii*. Detection method was not significantly associated with seroprevalence (*p* = 0.5952). The pooled estimates were 8.28% (95% CI: 5.69–11.89%) for ELISA, 7.74% (95% CI: 5.24–11.29%) for ELISA + WB, 7.50% (95% CI: 6.32–8.89%) for IFA, 12.91% (95% CI: 12.20–13.66%) for IFA + ELISA, 10.59% (95% CI: 8.60–12.97%) for IFA + WB, and 6.14% (95% CI: 2.53–14.14%) for WB (Supplementary File S9).

For PCR-based questing *Ixodes* tick studies, infection prevalence differed by geographical region (*p* = 0.0101). The highest pooled estimate was observed in Northeastern China (29.41%, 95% CI: 14.97–46.37%), whereas the lowest estimate was observed in Northern China (0.09%, 95% CI: 0.00–0.37%). No significant difference was observed across sampling years (*p* = 0.3723), with pooled estimates ranging from 14.45% (95% CI: 3.81–30.34%) in 1996–2005 to 30.83% (95% CI: 0.21–81.92%) in 2016–2025. Reported *Borrelia* genospecies was not significantly associated with tick infection prevalence (*p* = 0.1854), although the highest pooled estimate was observed for mixed detection of *B. garinii* and *B. afzelii* (32.64%, 95% CI: 14.01–54.71%). Tick species was also not significantly associated with infection prevalence (*p* = 0.1427); therefore, species-level estimates were interpreted descriptively, particularly because several subgroups were based on limited data and wide confidence intervals (Supplementary File S9).

## 4. Discussion

This systematic review and meta-analysis provides an updated synthesis of two epidemiologically different types of evidence in China: serological evidence of previous or cumulative exposure to *B. burgdorferi* sensu lato in humans, and PCR-confirmed infection in questing or unfed *Ixodes* ticks as an indicator of environmental hazard. The pooled human seroprevalence of *B. burgdorferi* sensu lato exposure was 7%, which was lower than the global estimate of 14.5% and lower than estimates reported for several high-prevalence regions, including Central Europe, Eastern Asia, and Western Europe [15]. However, comparisons between pooled estimates should be made cautiously because the included populations, exposure profiles, geographical coverage, and serological algorithms differed among reviews. A previous China-specific meta-analysis also showed that estimated seropositivity varied markedly according to whether studies used single-tier screening or confirmatory testing, indicating that pooled seroprevalence reflects not only true exposure but also the diagnostic criteria used to define positivity [16]. Therefore, the present estimate should be interpreted as evidence that exposure to *B. burgdorferi* sensu lato occurs at a non-negligible but heterogeneous level in China, rather than as a direct estimate of clinical Lyme borreliosis burden.

The pooled PCR-confirmed infection prevalence in questing or unfed *Ixodes* ticks was 20%, indicating that *B. burgdorferi* sensu lato circulates in host-seeking *Ixodes* tick populations in sampled habitats. This estimate should be interpreted as an indicator of environmental hazard rather than as a direct measure of human infection risk. Human seropositivity primarily reflects previous contact with *B. burgdorferi* sensu lato antigens and does not necessarily indicate active infection, current clinical disease, or the precise location where tick exposure occurred [17,18,19,20]. In contrast, PCR positivity in questing or unfed *Ixodes* ticks indicates the presence of infected host-seeking vectors at the sampling sites. Therefore, a high infection prevalence in questing ticks does not imply a proportionally similar level of human infection. Human exposure further depends on tick abundance, developmental stage, host-seeking activity, habitat type, human-biting activity, attachment duration, individual protective behavior, and the frequency with which people enter tick habitats [21,22].

Marked geographical heterogeneity was observed in human seroprevalence. Northeastern China had the highest pooled estimate, whereas lower estimates were observed in Eastern and Southwestern China. This pattern is epidemiologically plausible because northeastern China contains historically recognized natural foci of LB and overlaps with the distribution of *I. persulcatus*, an important vector of *B. burgdorferi* sensu lato in Asia [6,7,10]. The relatively high estimates reported for Heilongjiang and Inner Mongolia are also consistent with the presence of forest, forest-edge, and pastoral landscapes, where suitable microclimatic conditions, competent reservoir hosts, *Ixodes* vectors, and frequent human outdoor exposure may coexist [23,24,25]. Relatively high estimates in Xinjiang, Qinghai, Sichuan, Henan, and Jiangxi further suggest that human exposure is not restricted to the traditional northeastern foci. However, provincial estimates should be interpreted cautiously because many included studies were conducted in selected forests, endemic counties, hospitals, or occupational groups rather than in representative provincial populations.

Regional variation in human seroprevalence is unlikely to be explained by geography alone. Human exposure is determined by interactions among tick density, vector species, reservoir-host communities, vegetation, climate, land use, and the frequency and intensity of human contact with tick habitats [26,27,28]. Environmental suitability determines whether enzootic transmission can be maintained, whereas occupation, recreation, travel, and protective behavior determine whether humans actually encounter infected ticks. This may partly explain why some regions with relatively high tick infection prevalence showed only moderate human seroprevalence, whereas areas with moderate tick prevalence still showed substantial human exposure. Moreover, human antibodies may reflect exposure accumulated over several years or exposure acquired outside the place of residence, while tick infection prevalence reflects conditions at a specific collection site and time. Therefore, the spatial patterns observed in humans and ticks should be viewed as complementary rather than completely concordant evidence of LB exposure risk.

PCR-confirmed infection prevalence in questing or unfed *Ixodes* ticks also showed marked geographical heterogeneity. At the regional level, the highest pooled estimate was observed in Northeastern China, whereas lower estimates were observed in several other regions, particularly Northern China. This pattern suggests that the environmental hazard of *B. burgdorferi* sensu lato is concentrated in specific local enzootic foci rather than being uniformly distributed across China. This interpretation is epidemiologically plausible because the maintenance of *B. burgdorferi* sensu lato depends on the spatial overlap among competent *Ixodes* vectors, reservoir-host communities, suitable vegetation, and favorable microclimatic conditions [26,27]. It is also consistent with the highly heterogeneous tick fauna in China, where the distribution of medically important tick species is structured by climate, vegetation, host availability, and regional ecological zones [29,30]. In this context, the regional distribution of *I. persulcatus* provides a plausible vector-based explanation for the prominence of Northeastern China in the PCR-confirmed tick dataset [6,31]. Other *Ixodes* species, including *I. granulatus* and *I. sinensis*, have also been reported in more southern or ecologically heterogeneous settings, indicating that potentially relevant *Ixodes* habitats are not restricted to northeastern China [32]. However, regional or provincial tick infection prevalence should not be interpreted as a directly comparable measure of human disease risk. A high pooled estimate may reflect a localized natural focus, selected sampling sites, or intensive collection in known endemic areas, whereas human exposure additionally depends on the density and life stage of questing ticks, human-biting activity, protective behavior, and the frequency of human contact with infested habitats [21,22]. Therefore, the tick results should be interpreted primarily as indicators of environmental hazard, and future studies should combine PCR-based tick surveillance with data on tick density, reservoir hosts, human activity, and georeferenced environmental conditions.

Subgroup analyses by sampling period provide useful information, but they should be interpreted with caution. Human seroprevalence differed significantly among sampling periods, with a higher pooled estimate during 1996–2005 and a lower pooled estimate during 2016–2025. However, these period-specific estimates should not be interpreted as repeated temporal measurements from the same regions or populations. Instead, the studies included in each sampling period came from different combinations of provinces, study populations, and exposure settings, including forest areas, pastoral areas, hospitals, community populations, and occupationally exposed groups. Therefore, the observed differences among sampling periods may partly reflect changes in the geographical and population composition of the available studies, rather than a true national temporal trend. Earlier investigations often focused on forest, pastoral, and other occupationally exposed groups, which may have contributed to the relatively high estimates observed in some periods [33,34]. By contrast, the lower estimate during 2016–2025 may reflect differences in study location, target population, diagnostic approach, and surveillance context, rather than a definite decline in human exposure.

In questing or unfed *Ixodes* ticks, sampling period was not significantly associated with PCR-confirmed infection prevalence, and no consistent increasing or decreasing pattern was observed. This further suggests that the available data do not support a simple temporal trend in environmental circulation of *B. burgdorferi* sensu lato. Broader policy and diagnostic changes may provide historical context, but their effects could not be directly evaluated in this meta-analysis. China’s pesticide management framework was revised in 2001 and 2017, and associated changes in pesticide regulation and local pest-control practices may have influenced arthropod-control conditions [35]. However, their direct effects on tick abundance or *B. burgdorferi* sensu lato prevalence could not be determined from the included studies. Similarly, the national diagnostic standard for occupational Lyme disease introduced in 2019 may have influenced diagnostic recognition and case classification among exposed workers [36]. Overall, differences among sampling periods should be interpreted as period-stratified pooled estimates affected by study composition, geographical coverage, surveillance practices, diagnostic recognition, and environmental conditions, rather than as direct evidence of a national increase or decrease. For humans, the season of blood collection should also be interpreted cautiously, because antibody responses can persist for months or years and may not reflect the exact season of infection.

Neither antibody type nor serological detection method was significantly associated with human seroprevalence in univariate meta-regression. The pooled estimates for IgG, IgG + IgM, and IgM were broadly similar, although the IgM subgroup included fewer studies and showed greater uncertainty. This apparent similarity should not be interpreted as evidence that the antibody classes have the same biological meaning. IgM generally appears earlier after infection, whereas IgG develops later and may remain detectable for months or years after infection or treatment [17,18,19]. Because most included studies were cross-sectional and lacked information on symptom onset or previous treatment, each antibody subgroup probably contained a mixture of recent exposure, remote exposure, persistent antibody responses, and nonspecific reactivity. Human seroprevalence should therefore be interpreted primarily as evidence of previous or cumulative exposure rather than as the prevalence of active LB.

Seroprevalence varied descriptively among the included serological methods, but detection method was not significantly associated with human seroprevalence. Some combined-testing subgroups were represented by only a small number of studies, and their estimates should therefore not be used to rank diagnostic strategies. Moreover, the absence of a significant method effect does not demonstrate analytical equivalence. Broad method categories such as ELISA, IFA, WB, or combined testing strategies do not capture antigen composition, assay cutoff, immunoglobulin class, confirmation rules, disease stage, or background population. Clinical evaluations have shown that changing the sequence and composition of two-tier serological algorithms can improve sensitivity in early disease while maintaining high specificity [37,38]. Thus, part of the remaining heterogeneity may arise from variation within method categories that could not be captured by broad labels such as ELISA, IFA, or WB. Future epidemiological studies should report the complete testing algorithm, antigen platform, cutoff criteria, handling of equivocal results, and confirmatory procedure.

Age, sex, residence, and occupational category should be interpreted as indirect proxies of exposure rather than as determinants of susceptibility. Although age group was significantly associated with human seroprevalence, the pattern did not show a simple cumulative increase with age. This suggests that differences among age groups may be driven more by exposure opportunity, occupational composition, regional distribution, and outdoor activity patterns than by intrinsic age-related susceptibility. Similarly, the lack of a clear difference between men and women indicates that sex alone did not explain exposure in the pooled data. This does not mean that men and women had identical exposure patterns; rather, sex-related occupational and behavioral differences may vary by region and become diluted when heterogeneous populations are combined. Previous risk-factor analyses also suggest that specific outdoor activities and environmental contact are more informative predictors of tick-borne disease than demographic characteristics considered alone [11,39].

A reported history of tick bites provides a more direct clue to actual exposure. Participants reporting previous tick bites had higher seroprevalence than those without a reported bite history, supporting the biological link between recognized tick contact and exposure to *B. burgdorferi* sensu lato. However, tick-bite history should still be treated as an imperfect exposure indicator. Small immature ticks may remain unnoticed, tick bites may be forgotten, and the time interval between exposure and investigation may affect recall accuracy. In a clinical study, only a minority of children diagnosed with Lyme disease recalled a preceding tick bite, showing that absence of a recognized bite cannot reliably exclude previous exposure [40]. Therefore, the lower seroprevalence among participants without a reported tick bite should not be interpreted as evidence that they had no contact with ticks.

The exposure-risk and residence findings further show the limitations of broad population categories. The high-risk group had the highest pooled seroprevalence, which is consistent with repeated occupational or environmental contact with forested, grassland, pastoral, and tick-infested habitats. However, the low-risk estimate was higher than the moderate-risk estimate, indicating that these categories did not form a simple exposure–response gradient. This probably reflects the fact that occupation and residence do not fully capture the duration, frequency, season, and location of actual habitat contact. Individuals classified as low-risk may still be exposed through travel, recreation, gardening, collection of forest products, or visits to peri-urban green spaces, whereas persons in nominally high-risk occupations may reduce exposure through protective clothing, repellents, or routine tick checks. Rural residence may increase opportunities for contact with agricultural fields, forest margins, livestock, wildlife, and unmanaged vegetation, but residence is still only an indirect indicator of exposure. Recent epidemiological evidence indicates that hiking, walking, running, occupational activity in wooded or tall-grass environments, and time spent in residential yards can increase Lyme disease risk across rural, suburban, and urban settings [41]. Therefore, the type, intensity, and location of outdoor activity are likely more informative than residence or occupation alone. Finally, higher seroprevalence in environmentally exposed populations should not be interpreted as evidence that humans maintain the natural transmission cycle. Humans are incidental hosts; enzootic maintenance depends on competent reservoir hosts, *Ixodes* vectors, and suitable ecological conditions [24,25].

*Ixodes* species was not significantly associated with PCR-confirmed infection prevalence in the present analysis. Therefore, species-level estimates should be interpreted descriptively and should not be used to rank the epidemiological importance of different *Ixodes* species. Infection prevalence within a tick species is not equivalent to vector competence or public health relevance. A tick species with a high infection proportion may contribute little to human exposure if it is uncommon, geographically restricted, rarely bites humans, or lacks demonstrated transmission competence. Conversely, a species with a lower infection proportion may remain epidemiologically important if it is abundant, widely distributed, competent for transmission, and frequently encountered by humans. Experimental evidence has demonstrated transmission of *Borrelia garinii* by *I. persulcatus*, supporting its biological competence as a Lyme borreliosis vector [42]. Thus, species-specific risk assessment should integrate infection prevalence with tick abundance, developmental stage, host preference, human-biting behavior, geographical distribution, and direct evidence of vector competence.

Potential publication bias and small-study effects were detected among human serological studies, as indicated by funnel-plot asymmetry and Egger’s test. This suggests that smaller studies or studies reporting higher seroprevalence may have been more likely to be published or identified. However, the leave-one-out sensitivity analysis showed that the pooled human estimate was not driven by any single study, supporting the overall robustness of the finding. In contrast, no statistically significant funnel-plot asymmetry was detected among tick studies, although the smaller number of tick studies limited the power of this assessment.

This study has several limitations. First, substantial heterogeneity remained in both the human and tick analyses. This heterogeneity likely reflected differences in geographical region, study population, sampling design, diagnostic method, tick species, collection habitat, and laboratory protocol. Therefore, the pooled estimates should be interpreted as summaries of heterogeneous available evidence rather than precise national prevalence estimates for all populations or all questing *Ixodes* ticks in China.

Second, human seropositivity indicates previous or cumulative exposure rather than active infection or clinically confirmed Lyme borreliosis. Antibodies may persist for months or years, and the sampling site or place of residence may not represent the actual site or season of tick exposure. Therefore, provincial and seasonal patterns in human seroprevalence should be interpreted cautiously.

Third, subgroup analyses and meta-regression were based on aggregate study-level data and were conducted using univariate models. Potential correlations among region, sampling period, exposure group, diagnostic method, tick species, and habitat characteristics could not be controlled simultaneously. As a result, statistically significant associations should be interpreted as possible contributors to heterogeneity rather than independent causal effects. Some subgroups also contained few studies, which may have produced unstable estimates.

Finally, the available studies were unevenly distributed across China and many were conducted in selected endemic areas, occupational groups, hospitals, forest farms, or local tick habitats rather than through nationally representative sampling. In addition, standardized ecological information, including questing tick density, reservoir-host abundance, vegetation, climate, land use, and human activity, was often unavailable. The tick results therefore indicate environmental hazard but cannot directly quantify the density of infected ticks or the probability of human encounter with infected ticks. Future studies should integrate standardized human serosurveillance, PCR-based tick surveillance, tick-density data, reservoir-host information, and georeferenced environmental variables.

## 5. Conclusions

This systematic review and meta-analysis demonstrates that human exposure to *B. burgdorferi* sensu lato and PCR-confirmed infection in questing or unfed *Ixodes* ticks are widely documented but highly heterogeneous across China. Human seroprevalence varied by geographical region, sampling period, age group, reported tick-bite history, and exposure-risk category, whereas geographical region was the main source of heterogeneity in questing or unfed *Ixodes* ticks. These findings should be interpreted as evidence of heterogeneous exposure and environmental hazard rather than direct estimates of clinical Lyme borreliosis risk or independent causal effects.

The higher pooled prevalence observed in questing ticks does not imply an equivalent level of human infection, because human risk also depends on tick abundance, tick life stage, human-biting activity, habitat contact, and preventive behavior. Future studies should use standardized human serological algorithms, consistent tick-collection and PCR-detection protocols, and integrated surveillance of humans, questing ticks, reservoir hosts, and environmental conditions. A One Health surveillance framework will be important for improving risk assessment and guiding Lyme borreliosis prevention in China.

## Figures and Tables

**Table 1:** Provincial prevalence coefficients for *Borrelia burgdorferi* sensu lato infection in humans and questing *Ixodes* ticks in China.

Host	Province	Prevalence	Coefficient (95% CI)	Host	Province	Prevalence	Coefficient (95% CI)
**HUMANS**	Zhejiang	0.0630	[0.0276–0.1114]	Ticks	Zhejiang	0.0460	[0.0286–0.0672]
	Yunnan	0.0705	[0.0113–0.1744]		Yunnan	0.1609	[0.0917–0.2450]
	Xinjiang	0.1036	[0.0721–0.1401]		Xinjiang	0.4000	[0.2755–0.5314]
	Tibet	0.0363	[0.0033–0.1023]		Tianjin	0.0000	[0.0000–0.0105]
	Tianjin	0.0587	[0.0491–0.0692]		Shaanxi	0.8250	[0.6936–0.9254]
	Sichuan	0.1245	[0.0456–0.2348]		Qinghai	0.2271	[0.1727–0.2865]
	Shanxi	0.0485	[0.0261–0.0772]		Ningxia	0.4545	[0.1848–0.7397]
	Shanghai	0.0166	[0.0116–0.0225]		Jilin	0.2171	[0.0731–0.4106]
	Shandong	0.0626	[0.0575–0.0678]		Inner Mongolia	0.1090	[0.0440–0.1984]
	Shaanxi	0.0483	[0.0188–0.0905]		Hunan	0.1293	[0.0801–0.1881]
	Qinghai	0.1507	[0.1361–0.1659]		Heilongjiang	0.3531	[0.1319–0.6144]
	Liaoning	0.0744	[0.0530–0.0991]		Guizhou	0.2063	[0.1615–0.2551]
	Jilin	0.0643	[0.0426–0.0900]		Gansu	0.0377	[0.0001–0.1388]
	Jiangxi	0.1244	[0.0254–0.2831]		Beijing	0.0011	[0.0000–0.0042]
	Jiangsu	0.0414	[0.0185–0.0728]				
	Inner Mongolia	0.1091	[0.0666–0.1606]				
	Hunan	0.0749	[0.0629–0.0879]				
	Hubei	0.0989	[0.0854–0.1132]				
	Henan	0.1171	[0.0805–0.1594]				
	Heilongjiang	0.2366	[0.1308–0.3623]				
	Hebei	0.0266	[0.0000–0.0995]				
	Hainan	0.0784	[0.0445–0.1208]				
	Guizhou	0.0453	[0.0035–0.1309]				
	Guangxi	0.0152	[0.0076–0.0255]				
	Guangdong	0.0555	[0.0211–0.1049]				
	Gansu	0.0819	[0.0704–0.0942]				
	Fujian	0.0229	[0.0169–0.0299]				
	Beijing	0.0760	[0.0391–0.1238]				
	Anhui	0.0787	[0.0137–0.1902]				

CI—confidence interval.

## Data Availability

The original contributions presented in this study are included in the article and Supplementary Materials, including the study-level dataset provided as Supplementary File S10. Further inquiries can be directed to the corresponding author.

## Supplementary Materials

The following supporting information can be downloaded at https://www.mdpi.com/article/10.3390/microorganisms14071563/s1, Supplementary File S1 Table S1: Retrieval formulas in six databases in present study; Supplementary File S2: References for the human and tick studies included in the systematic review and meta-analysis; Supplementary File S3 Figure S1: Forest plot of *Borrelia burgdorferi* sensu lato seroprevalence in humans and tick in China; Supplementary File S4 Figure S2: Funnel plots of *B. burgdorferi* sensu lato seroprevalence in humans and infection prevalence in questing *Ixodes* ticks in China; Supplementary File S5 Figure S3: Trim-and-fill-adjusted funnel plots of *Borrelia burgdorferi* sensu lato seroprevalence in humans and infection prevalence in questing *Ixodes* ticks in China; Supplementary File S6 Figure S4: Egger’s regression plots assessing small-study effects in studies of *Borrelia burgdorferi* sensu lato prevalence in China; Supplementary File S7 Figure S5: Leave-one-out sensitivity analysis of *Borrelia burgdorferi* sensu lato seroprevalence in humans in China; Supplementary File S8 Figure S6: Leave-one-out sensitivity analysis of *Borrelia burgdorferi* sensu lato PCR-confirmed infection prevalence in questing or unfed *Ixodes* ticks in China; Supplementary File S9 Table S2: Summary of subgroup analyses and univariate meta-regression for human seroprevalence and PCR-confirmed infection prevalence in questing or unfed *Ixodes* ticks in China; Supplementary File S10: Study-level data extracted from the included human and questing or unfed *Ixodes* tick studies, including the data used in the subgroup analyses.
