# Supplementary material for: Prevalence and Associated Factors of Borrelia burgdorferi Sensu Lato Exposure in Humans and Infection in Questing Ixodes Ticks in China: A Systematic Review and Meta-Analysis
Source: Microorganisms. 2026 Jul 17;14(7):1563. doi: 10.3390/microorganisms14071563 (PMC13413532; doi:10.3390/microorganisms14071563)
Supplement: Supplementary file 1 [file microorganisms-14-01563-s001.zip › Supplementary Materials.pdf]

microorganisms

MDPI

Supplementary File S1

Table S1. Retrieval formulas in six databases in present study.

Database	Search Details
PubMed 160	(Lyme disease OR Borrelia burgdorferi) AND (human OR people OR person OR man OR men OR women OR woman OR patient OR Ixodes tick) AND (infection rate OR prevalence OR seroprevalence OR serological survey OR sero-prevalence OR seroepidemiology OR sero-epidemiology OR prevalence OR epidemiology) AND (China OR chinese)
Web of Science 178	#1<br>TS=(Lyme disease OR *Borrelia burgdorferi*)<br>#2<br>TS= (human OR people OR person OR man OR men OR women OR woman OR patient OR Ixodes tick)<br>#3<br>TS=(infection rate OR prevalence OR seroprevalence OR serological survey OR sero-prevalence OR seroepidemiology OR sero-epidemiology OR prevalence OR epidemiology)<br>#4<br>TS=(China OR chinese)<br>Final query = #1 AND #2 AND #3 AND #4
ScienceDirect 1068	(Lyme disease OR Borrelia burgdorferi) AND (human OR people OR person OR Ixodes tick) AND (China OR chinese)
CNKI 260	(Lyme disease OR *Borrelia burgdorferi*) and (humans OR Ixodes ticks) (In chinese)
Wanfang 679	(Lyme disease OR *Borrelia burgdorferi*) and (humans OR Ixodes ticks) (In chinese)
Vip 220	(Lyme disease OR *Borrelia burgdorferi*) and (humans OR Ixodes ticks) (In chinese)

Supplementary File S2. References for the human and tick studies included in the systematic review and meta-analysis.

1. Ai, C.X.; Wen, Y.X.; Zhang, Y.G.; Wang, S.S.; Qiu, G.C.; Shi, Z.X.; Chen, Z.Q.; Li, D.Y.; Liu, X.D. Epidemiological investigation of Lyme disease in the forest area of Hailin County, Heilongjiang Province. *Chin. J. Public Health* **1987**, (2), 82–85, 128. (In Chinese)
2. Ai, C.X.; Wen, Y.X.; Zhang, Y.G.; Wang, S.S.; Qiu, Q.C.; Shi, Z.X.; Li, D.Y.; Chen, D.Q.; Liu, X.D.; Zhao, J.H. Clinical manifestations and epidemiological characteristics of Lyme disease in Hailin county, Heilongjiang Province, China. *Ann. N. Y. Acad. Sci.* **1988**, 539, 302–313.
3. Ai, C.X.; Zhang, W.F.; Zhao, J.H. Sero-epidemiology of Lyme disease in an endemic area in China. *Microbiol. Immunol.* **1994**, 38(7), 505–509.
4. Bai, X.J. Seroepidemiological survey of Lyme disease in Tianzhu County, Gansu Province. *J. Prev. Med. Chin. PLA* **1997**, (6), 65. (In Chinese)
5. Cai, D.H.; He, Y.X.; Wang, J.S.; Fu, Y.W.; Yu, D.S.; Cai, Y.H.; Zhang, D.C.; Zhang, Z.Q.; Sun, G.J. Investigation of a natural focus of Lyme disease in Qingyuan County. *Chin. J. Vector Biol. Control* **1994**, (5), 382–383. (In Chinese)
6. Cai, S.L.; Duan, Z.X.; Qu, S.H. Seroepidemiological survey of Lyme disease among residents of Huairou District, Beijing. *Chin. J. Vector Biol. Control* **2003**, (6), 424. (In Chinese)
7. Cao, C.J.; Li, H.; Wang, Q.; Yang, L.Q.; Yuan, L.F.; Tian, J.H.; Cao, X.; Gao, L.H.; Zhang, J.H.; Zhang, Z.F.; et al. Serological and symptomatological survey of Lyme disease in the Greater Khingan forest area of Inner Mongolia. *Chin. J. Zoonoses* **1997**, (4), 76–77. (In Chinese)
8. Cao, W.C.; Zhao, Q.M.; Zhang, P.H.; Dumler, J.S.; Zhang, X.T.; Fang, L.Q.; Yang, H. Granulocytic Ehrlichiae in *Ixodes persulcatus* ticks from an area in China where Lyme disease is endemic. *J. Clin. Microbiol.* **2000**, 38(11), 4208–4210.
9. Cao, W.C.; Zhao, Q.M.; Zhang, P.H.; Yang, H.; Wu, X.M.; Wen, B.H.; Zhang, X.T.; Habbema, J.D. Prevalence of *Anaplasma phagocytophila* and *Borrelia burgdorferi* in *Ixodes persulcatus* ticks from northeastern China. *Am. J. Trop. Med. Hyg.* **2003**, 68(5), 547–550.
10. Chen, W.S.; Hao, R.F.; Luo, H.M.; Li, W.; He, J.F.; Zheng, K. Seroepidemiological study of Lyme disease in Guangdong Province. *Dis. Surveill.* **1999**, 14(11), 423–425. (In Chinese)
11. Chen, Y.L.; Yang, Y.S.; Jia, L.L.; Geng, L.B.; Wang, Y.Q.; Zheng, F.H. Seroepidemiological survey of Lyme disease in Miyun County, Beijing. *Cap. J. Public Health* **2016**, 10(2), 83–85. (In Chinese)
12. Chu, C.Y.; He, J.; Wang, J.B.; Hasengaowa; Zhang, P.H.; Wu, X.M.; Zhao, Q.M.; Jiang, B.G.; Gao, Y.; Cao, W.C. Infection and genotyping of Lyme borreliae in ticks and rodents from the Greater Khingan forest area. *Chin. J. Epidemiol.* **2006**, 27(8), 681–684. (In Chinese)
13. Cui, W.W.; Deng, J.; Shi, S.F.; Huang, R.J.; Xu, K.; Huang, C.X.; Gan, W.Q.; Xu, X.P.; Xu, X.J.; Sun, Z.; et al. Seroepidemiological survey of Lyme disease in Hangzhou. *Chin. Prev. Med.* **2007**, (4), 382–386. (In Chinese)
14. Dong, J.H.; Zhu, J.H.; Yin, F.R. Survey of Lyme disease prevalence among residents of the Greater Khingan forest area. *Pract. Prev. Med.* **2007**, (5), 1457–1458. (In Chinese)
15. Dou, X.; Lyu, Y.; Jiang, Y.; Tian, L.; Li, X.; Lin, C.; Sun, Y.; Guan, Z.; Zhang, X.; Wang, Q. Seroprevalence of Lyme disease and associated risk factors in rural population of Beijing. *Int. J. Clin. Exp. Med.* **2015**, 8(5), 7995–7999.

16. Duan, X.D.; He, Z.H.; Gao, Z.H.; Jiang, B.G.; Gong, Z.D.; Zhang, Y.; Shao, Z.T.; Jiang, J.F.; Sun, Y.; Liu, H.B.; et al. Detection and identification of *Borrelia sinica* in *Ixodes ovatus* from the border region of Yunnan Province. *Chin. J. Vector Biol. Control* **2019**, 30(5), 519–523. (In Chinese)
17. Du, L.Y.; Chen, X.N.; Sun, Y.; Guo, T.Y.; Lu, L.; Zhang, P.H. Serological survey of Lyme disease in the forest area of Chengde, Hebei Province. *J. Chengde Med. Coll.* **2005a**, (1), 14–16. (In Chinese)
18. Du, L.Y.; Chen, X.N.; Sun, Y.; Guo, T.Y.; Lu, L.; Zhang, P.H. Investigation of the principal biological vectors of Lyme disease in the Chengde forest area. *Chin. J. Zoonoses* **2005b**, 21(6), 534–536. (In Chinese)
19. Du, Y. Investigation report on Lyme disease in the Weihe forest area of Heilongjiang Province. *Chin. J. Zoonoses* **1991**, (4), 36–37. (In Chinese)
20. Du, Y.H.; Zhao, J.Y.; Lu, X.; Bai, J.M.; Xia, S.L. Preliminary seroepidemiological survey of Lyme disease in Henan Province. *Mod. Prev. Med.* **2012**, 39(14), 3681–3682. (In Chinese)
21. Fang, H.L.; Wang, J.J.; Huang, H.; Duan, X.M. Epidemiological investigation of Lyme disease-associated myocardial injury in the Mangshan forest area of southern Hunan. *Chin. Gen. Pract.* **2010**, 13(9), 984–985. (In Chinese)
22. Fan, L.H.; Wang, L.S.; Zou, H.Y.; Chang, S.W.; Wang, Y.Q. Seroepidemiological survey of Lyme disease at forest farms in the Heihe region. *Chin. J. Vector Biol. Control* **2002**, (5), 347. (In Chinese)
23. Fan, Z.B.; Gao, X.; Zhang, Y.Y.; Chen, Z.H.; Zhang, W. Epidemiological and clinical characteristics of tick-borne diseases over the past decade at an infectious disease hospital in Beijing. *Chin. J. Zoonoses* **2024**, 40(4), 315–322. (In Chinese)
24. Feng, B.Q.; Chen, W.S.; Du, Z.M.; Luo, H.M.; Hou, X.X.; Wan, K.L.; Zhang, Z.F. Results of a Lyme disease investigation in Guangdong Province in 1992. *Guangdong Health Epidemic Prev.* **1993**, (4), 7–8. (In Chinese)
25. Feng, F.B.; Zhang, W.F.; Zhou, G.P.; Zhang, S.Y.; Yuan, Y.M.; Wu, Y.S. Discovery and clinical study of Lyme disease in the Beijing region. *Chin. J. Epidemiol.* **1994**, 15(1), 10–13. (In Chinese)
26. Gao, D.Q.; Cao, W.C.; Zhang, X.T.; Zhao, Q.M.; Zhu, J.H. Investigation of a natural focus of human ehrlichiosis in the Greater Khingan forest area of Inner Mongolia. *Infect. Dis. Inf.* **2001**, (4), 168–170. (In Chinese)
27. Gao, D.Q.; Cao, W.C.; Zhang, X.T.; Sun, Y.; Zhang, P.H.; Zhu, J.H. Investigation of risk factors for Lyme borrelia infection and tick-prevention measures among residents of the Greater Khingan forest area. *J. Med. Pest Control* **2001**, (1), 22–25. (In Chinese)
28. Gaowa; Wulantuya; Sato, K.; Liu, D.; Cui, Y.; Yin, X.; Zhang, L.; Li, H.; Wang, T.; Liu, R.; et al. Surveillance of *Borrelia miyamotoi*-carrying ticks and genomic analysis of isolates in Inner Mongolia, China. *Parasit. Vectors* **2021**, 14(1), 368.
29. Ge, H.; Yue, J.N.; Qin, Y.Q.; Shi, Y. Investigation of Lyme disease in selected areas of Qinghai Province. *Chin. J. Vector Biol. Control* **2008**, (2), 120. (In Chinese)
30. Geng, Z.; Hou, X.X.; Guo, J.H.; Huang, X.; Wang, C.S.; Wang, B.; Yang, X.J.; Hao, Q. Epidemiological investigation of Lyme disease in Changbai and Tonghua counties, Jilin Province. *Chin. J. Vector Biol. Control* **2010a**, 21(6), 572–575. (In Chinese)
31. Geng, Z.; Hou, X.X.; Wan, K.L.; Hao, Q. Isolation and identification of Lyme borreliae from ticks in six provinces of China. *Chin. J. Epidemiol.* **2010b**, (12), 1346–1348. (In Chinese)
32. Gong, Z.W.; Shi, S.Z.; Luo, Y.Q.; Yang, X.M.; Wang, C.D.; Shi, H.Y. Preliminary investigation of Lyme disease in a military unit and its surrounding area in Jiuquan. *Lanzhou Sci. Technol. Inf.* **1997**, (4), 10–11. (In Chinese)

33. Gong, Z.Y.; Jiang, L.P.; Wang, Z.; Gao, Y.; Fang, C.F.; Wang, W.; Cui, Q.R.; Ge, J.H.; Pang, W.L.; Chen, Y.J.; et al. Preliminary seroepidemiological survey of Lyme disease in Zhejiang Province. *Dis. Surveill.* **2005**, (10), 510–512. (In Chinese)

34. Guan, R.; Wei, N.; Cao, M.; Yang, J.G.; Wu, R.; Li, H. Prevalence of three important tick-borne pathogens in ticks and humans in Shaanxi Province, Northwest China. *Parasitol. Int.* **2025**, 104, 102980.

35. Guan, S.Z.; Yin, G.C.; Han, S.Q.; Hua, B.; Liu, Q.Y. Detection of serum antibodies against *Borrelia burgdorferi* using the VIDAS immunoassay system. *Chin. J. Epidemiol.* **1994**, (6), 368–371. (In Chinese)

36. Gu, C.G.; Li, Y.J.; Pan, B.S. Detection of antibodies against Lyme borreliae among residents of the Heihe forest area using ELISA. *Heilongjiang Med. Pharm.* **2003**, (1), 63. (In Chinese)

37. Gu, C.G.; Cao, X.H.; Jia, Y.P.; Yang, S.J.; Kong, J.S. Seroepidemiological survey of Lyme disease in selected forest areas of the Lesser Khingan Mountains. *Mod. Prev. Med.* **2014**, 41(6), 1125–1126, 1129. (In Chinese)

38. Guo, W.S.; Li, L.C.; Xia, Z.G.; Zhang, Y.P.; Guo, F.Z.; Shang, S.Y. Seroepidemiological survey of Lyme disease in Xin'an County. *Henan J. Prev. Med.* **1994**, (3), 139–140. (In Chinese)

39. Guo, Y.; Wan, K.L.; Xu, S.E.; Zhang, Z.F.; Xie, L.C.; Chen, S.B.; Pan, L.X. Discovery and investigation of a Lyme disease focus in eastern Guangdong. *Chin. J. Zoonoses* **2000**, (2), 42–45. (In Chinese)

40. Hao, Q.; Yang, X.J.; Hou, X.X.; Wang, C.S.; Geng, Z.; Wang, Y.H.; Du, Z.S.; Wan, K.L. Etiological investigation and genotyping of Lyme disease in Jilin Province. *Chin. J. Vector Biol. Control* **2007**, 18(4), 303–305. (In Chinese)

41. Hao, Q.; Geng, Z.; Hou, X.X.; Tian, Z.; Yang, X.J.; Jiang, W.J.; Shi, Y.; Zhan, Z.F.; Li, G.H.; Yu, D.S.; et al. Seroepidemiological investigation of Lyme disease and human granulocytic anaplasmosis among people living in forest areas of eight provinces in China. *Biomed. Environ. Sci.* **2013**, 26(3), 185–189.

42. He, H.; Tian, H. First seroepidemiological survey of Lyme disease at Guiyang Airport. *Guizhou Med. J.* **1995**, (6), 349–350. (In Chinese)

43. He, L.F.; Hou, X.X.; Chen, T.; Zhang, L.; Wen, S.; Miao, G.Q.; Xing, M.; Hao, Q.; Zhu, X. Serological study of Lyme disease antibodies in 2,311 patients with arthritis symptoms in Hainan Province. *Chin. J. Prev. Med.* **2021**, 55(3), 379–385. (In Chinese)

44. Hou, J.; Ling, F.; Chai, C.; Lu, Y.; Yu, X.; Lin, J.; Sun, J.; Chang, Y.; Ye, X.; Gu, S.; et al. Prevalence of *Borrelia burgdorferi* sensu lato in ticks from eastern China. *Am. J. Trop. Med. Hyg.* **2015**, 92(2), 262–266.

45. Hou, X.X.; Liu, Y.; Hao, Q.; Chen, J.Y.; Geng, Z.; Song, C.Y.; Chen, J.; Ding, J.Q.; Yu, F.T.; Jia, Y.H.; et al. Investigation of the principal biological vectors of Lyme borreliae in Jixian County, Tianjin. *Chin. Prev. Med.* **2008**, 9(5), 358–359. (In Chinese)

46. Hua, M.T.; Lin, T.; Liu, Z.L.; Jin, Z.Q.; Zhang, Z.F.; He, C.; Wang, Z.T.; Yang, X.K. Serological survey of Lyme disease in humans and livestock in the Altay region of Xinjiang. *Med. J. Natl. Defending Forces Northwest China* **1998**, (3), 47–48. (In Chinese)

47. Hua, M.T.; Jin, Z.Q.; Lin, T.; Gong, Z.W.; He, C.; Liu, Z.L. Investigation of natural foci of Lyme disease along the China-Russia and China-Kazakhstan border regions. *J. Prev. Med. Chin. PLA* **1999**, (6), 402–405. (In Chinese)

48. Hua, M.T.; Liu, Z.L.; Wang, K.; Fan, Y.C.; Lu, H.B.; Jin, Z.Q. Detection of serum antibodies against Lyme disease among military personnel and local residents in the Altay mountain forest area. *Med. J. Natl. Defending Forces Northwest China* **2002**, (1), 66–67. (In Chinese)

49. Huang, H.N.; Ding, Z.; He, J.; Wu, X.M.; Jiang, B.G.; Gao, Y.; Zhao, Q.M.; Wang, Y.F.; Cao, W.C. Investigation of Lyme borrelia infection in animals from forest areas of Jilin Province. *Chin. J. Zoonoses* **2006a**, 22(8), 785–788. (In Chinese)

50. Huang, H.N.; Ding, Z.; He, J.; Wu, X.M.; Jiang, B.G.; Gao, Y.; Cao, W.C. Investigation of Lyme borrelia infection in animals from forest areas of Jilin Province. *Chin. J. Zoonoses* **2006b**, (8), 785–788. (In Chinese)

51. Huang, X.M.; He, H.J.; Yang, Z.Y.; Ou, L.M.; Zhang, X.Q.; Liu, Q.W.; Huang, H. Investigation of a Lyme disease focus in the Mangshan forest area of southern Hunan. *Pract. Prev. Med.* **2000**, (6), 415–417. (In Chinese)

52. Huang, Y.P.; Zheng, H.N.; Cai, S.G.; Shi, L.S.; Pan, L.; Huang, X.H.; Yu, E.S. Discovery and investigation of Lyme disease in southern Fujian Province. *Chin. J. Zoonoses* **1993**, (1), 46–47. (In Chinese)

53. Huang, Z.Y.; Hou, X.X.; Wan, K.L. Investigation of Lyme disease in Pingjiang County, Hunan Province. *Chin. J. Vector Biol. Control* **2001**, (4), 293–294. (In Chinese)

54. Hu, S.S.; Zhang, D.R.; Wang, C.J.; Wu, C.J.; Wan, Z.D.; Wei, Q.; Wei, G.X. Investigation of Lyme disease in Lu'an, Anhui Province. *Chin. J. Zoonoses* **1999**, (4), 113–114. (In Chinese)

55. Jia, W.C.; Wan, K.L.; Zhang, X.J.; Zhang, Z.F.; Zhang, S.Q.; Jia, Z.B.; Hui, G.L.; Song, H.Z.; Mu, J.W. Investigation of Lyme disease in the Lesser Khingan forest area of Heilongjiang Province. *Chin. J. Vector Biol. Control* **1998**, (4), 39–41. (In Chinese)

56. Jia, Y.P.; He, J.H.; Gao, F.; Li, Y.Q.; Zhang, X.H.; Xie, Z.C. Analysis of Lyme disease infection in a border-defense unit in Inner Mongolia. *J. Prev. Med. Chin. PLA* **2002**, (2), 123–124. (In Chinese)

57. Ji, C.L. Investigation of a tick-borne Lyme disease focus in the Alatanheli area of Bogda Mountain. *J. Clin. Med. Lit. Electron. Ed.* **2017**, 4(45), 8886, 8888. (In Chinese)

58. Ji, H.; Chang, L.; Yan, Y.; Sun, H.; Wang, L. Pilot Surveillance of *Babesia*, *Borrelia burgdorferi*, and *Anaplasma phagocytophilum* Among Chinese Blood Donors - Xinjiang, Inner Mongolia, and Heilongjiang PLADs, China, 2022-2023. *China CDC Wkly.* **2024**, 6(46), 1206–1210.

59. Lai, C.L.; Fang, J.S.; Ceng, J.; Li, Y.L.; Zheng, Q.Q.; Zhang, Y.S.; Xian, G.H.; Wang, S.S. Lyme disease investigation in Guangxi I: Preliminary seroepidemiological survey of 457 forest-farm workers. *Guangxi Med. J.* **1991**, (5), 343–344. (In Chinese)

60. Lai, C.L.; Fang, J.S.; Liu, J.; Huang, F.X.; Li, Y.; Li, T.G. Serological survey of Lyme disease among patients in three psychiatric hospitals. *Guangxi J. Prev. Med.* **1995**, (3), 168. (In Chinese)

61. Li, H.; Zhang, X.G.; Yan, D.C.; Li, F.K. Analysis of Lyme borrelia infection among psychiatric patients. *Chin. J. Vector Biol. Control* **1998**, (4), 42–43. (In Chinese)

62. Li, H.; Li, D.J.; Zhang, C.F. Preliminary study of the relationship between Lyme borrelia infection and mental disorders. *Chin. J. Zoonoses* **1999**, (2), 6. (In Chinese)

63. Li, H.; Zhang, X.G.; Xin, Z.L.; Wang, Q.; Wang, Y. Investigation of the epidemiological status of Lyme disease in the Greater Khingan forest area of Inner Mongolia. *Chin. J. Epidemiol.* **2003c**, 24(8), 733. (In Chinese)

64. Li, H.B.; Hu, L.M.; Wei, A.M.; Zhang, Z.Q.; Liu, H.; Lu, Z.X. Survey of antibodies against several tick-borne infectious diseases among residents of selected areas in the three northeastern provinces of China. *Med. J. Shenyang Mil. Reg.* **2003a**, (5), 371–372. (In Chinese)

65. Li, H.Y.; Zhao, S.S.; Zhang, L.; Zhang, K.; Chen, C.F.; Wang, Y.Z. Molecular epidemiological survey of *Anaplasma* and *Borrelia* in parasitic ticks from northern Xinjiang. *J. Shihezi Univ. Nat. Sci.* **2017**, 35(1), 108–112. (In Chinese)

66. Li, L.C.; Guo, W.S.; Zhang, Y.P.; Xia, Z.G.; Guo, F.Z.; Chen, K.L.; Yue, M.J.; Zhou, Q.C. Seroepidemiological survey of Lyme disease in Henan Province. *Henan Med. Res.* **1994**, 3(2), 159–161. (In Chinese)
67. Lin, C.H. Serological survey of Lyme disease in the Changbai Mountain forest area. *J. Med. Sci. Yanbian Univ.* **2008**, 31(4), 272–274. (In Chinese)
68. Lin, Y.L.; Zhang, W.L.; Duojiouzhu; He, Z.; Lu, Z.H.; Cirenpanduo; Zhao, Z.; Li, L.; Long, Y. Molecular identification of ticks and investigation of tick-borne pathogens in Yadong County, Tibet. *Chin. J. Zoonoses* **2023**, 39(12), 1239–1246. (In Chinese)
69. Li, S.; Chen, T.; Li, H.; Yu, S.Y.; Chen, H.; Zhu, X. Serological screening for Lyme disease among patients with neurological symptoms in Sanya. *Lab. Med. Clin.* **2018**, 15(10), 1465–1467. (In Chinese)
70. Li, S.; Zhang, L.; Li, H.; Hou, X.X.; Chen, T.; Miao, G.Q.; Fu, X.Y.; Chen, H.; Hao, Q.; Zhu, X. Analysis of Lyme disease antibody test results among a group of clinic patients in northeastern Hainan Province. *Pract. Prev. Med.* **2020**, 27(9), 1068–1071. (In Chinese)
71. Liu, F.Q.; Hao, Q.; Gao, L.D.; Geng, Z.; Zhan, Z.F.; Hou, X.X.; Zhang, H.; Wang, M.W.; Li, J.H.; Guo, S.H.; et al. Preliminary investigation of the epidemiological status of Lyme disease in two mountainous townships of Hunan Province. *Dis. Surveill.* **2008**, (6), 337–340. (In Chinese)
72. Liu, M.S.; Wu, Y.J.; Zhao, Z.F. Preliminary serological survey of Lyme disease among clinic attendees in Changzhi, Shanxi Province. *J. Changzhi Med. Coll.* **2006**, (1), 17–18. (In Chinese)
73. Liu, Q.W.; Zhang, L.X.; Li, J.H.; Wan, K.L.; Yang, L.; Tao, X.Y.; Xu, Q.S. Seroepidemiological survey of Lyme disease in Chenzhou. *Dis. Surveill.* **1997a**, (5), 32–33. (In Chinese)
74. Liu, Q.W.; Li, J.H.; Zhang, L.X.; Wan, K.L.; Yang, L.; Tao, X.Y.; Xu, Q.S. Investigation of Lyme disease in Chenzhou, Hunan Province. *Chin. J. Vector Biol. Control* **1997b**, (2), 125–126. (In Chinese)
75. Liu, S.L.; Wang, L.J.; Wan, K.L.; Lin, H.Q.; Chen, Z.L.; Hou, X.X. Investigation of a natural focus of Lyme disease in the Yimeng mountain area of Shandong Province. *Chin. J. Vector Biol. Control* **2000a**, (3), 194–196. (In Chinese)
76. Liu, Y.; Tan, Y.H.; Sun, H.; Li, J.; Yan, S.S.; Hou, X.X.; Wan, K.L. Seroepidemiological survey of Lyme disease at Shawan Forest Farm, Xinjiang. *Chin. J. Vector Biol. Control* **2002**, (1), 53–54. (In Chinese)
77. Liu, Y.; Geng, Z.; Chen, J.Y.; Hou, X.X.; Song, C.Y.; Hao, Q.; Yu, F.T.; Zhang, Z.F.; Jia, Y.H.; Zhao, Z.W.; et al. Isolation of Lyme borreliae from blood of patients with polyneuritis in Jixian County, Tianjin. *Tianjin Med. J.* **2008**, (5), 345. (In Chinese)
78. Liu, Z.J.; Shi, S.Z.; Wang, D.H.; Zhang, J.; Yang, Y.S.; Chen, Y.K.; Li, J.H.; Yang, D.L. Seroepidemiological survey of human Lyme disease in the Diebu forest area. *Chin. J. Public Health* **1994**, (2), 68. (In Chinese)
79. Liu, Z.J.; Hua, M.T.; Shi, S.Z.; Yang, Y.S.; Luo, Y.Q.; Gong, Z.W. Study of the relationships of Lyme disease with humans, domestic animals, and rodents in parts of northwestern China. *J. Med. Pest Control* **2000b**, (6), 298–301. (In Chinese)
80. Liu, Z.J.; Sun, Y.; Shi, S.Z.; Gong, Z.W.; Zhang, J.J.; Xu, R.M.; Lu, B.L. Investigation of a natural focus of Lyme disease in the Lazikou forest area. *Chin. J. Zoonoses* **2004**, (5), 445–447. (In Chinese)
81. Liu, Z.J.; Shi, S.Z.; Zhang, J.J.; Gong, Z.W. Investigation of coinfection with Lyme disease, tularemia, scrub typhus, and Q fever in the Diebu forest area. *World J. Infect.* **2005**, 5(1), 45–47. (In Chinese)
82. Liu, Z.Y.; Hou, X.X.; Huo, Q.B.; Geng, Z.; Liu, J.; Wan, K.L.; Hao, Q. Comparison of IFA, ELISA, and western blotting for serological diagnosis of Lyme disease. *Chin. J. Vector Biol. Control* **2011**, 22(3), 236–238. (In Chinese)

83. Liu, Z.Y.; Hao, Q.; Hou, X.X.; Jiang, Y.; Geng, Z.; Wu, Y.M.; Wan, K.L. A study of the technique of western blot for diagnosis of Lyme disease caused by *Borrelia afzelii* in China. *Biomed. Environ. Sci.* **2013**, 26(3), 190–200.

84. Li, X.M.; Zhang, D.R.; Chen, C.W.; Zhang, Y.; Fang, G.Q.; Zhang, C.; Zheng, Y. Seroepidemiological survey of Lyme disease in Hongta District, Yuxi City. *Chin. J. Vector Biol. Control* **2000**, (1), 15. (In Chinese)

85. Li, Y.L.; Hao, J.G.; Zhang, Z.F.; Wan, K.L.; Xia, X.Z.; Zhang, J.S.; Hu, X.W.; Liu, H.; Jiang, M.Z.; Feng, F.L. Investigation of Lyme disease in Nanchuan County, Sichuan Province. *Chin. J. Vector Biol. Control* **1991**, (1), 54–56. (In Chinese)

86. Li, Y.T.; Cao, Y.L.; Shi, J.M.; Lu, D.X.; Shen, W.J.; Sun, H.Z.; Li, B.Q. Seroepidemiological survey of Lyme disease in Shanghai. *Chin. J. Vector Biol. Control* **1996**, 7(1), 59–60. (In Chinese)

87. Li, Y.X.; Jiang, H.H.; Chen, Y.L.; Zhang, Y.W.; Ma, D.H.; Wang, F.C. Lyme disease presenting mainly with facial paralysis: A seroepidemiological survey and report of two cases. *J. Bengbu Med. Coll.* **1995**, (5), 327–328. (In Chinese)

88. Li, Z.Q.; Gong, Z.W.; Fei, J.X.; Shi, S.G.; Liu, T.; Feng, X.Y.; Yang, G. Seroepidemiological investigation of Lyme disease in Shaanxi Province. *Shaanxi Med. J.* **2010**, 39(3), 352–353. (In Chinese)

89. Li, Z.W.; Wang, J.B.; Yuan, H.Y.; Tian, C.Y. Seroepidemiological analysis of asymptomatic Lyme disease infection. *J. Med. Pest Control* **2003b**, (12), 758–759. (In Chinese)

90. Long, J.; Lin, T.; Li, W.B.; Zhang, Z.F. Epidemiological investigation of Lyme disease in humans and animals in Shanggao County, Jiangxi Province. *Chin. J. Vector Biol. Control* **1999**, (1), 51–53. (In Chinese)

91. Lu, J.P.; Ye, Z.X.; Yang, X.F.; Li, Q.A.; Han, X.Z.; Zhang, Z.F.; Wang, H.Y.; Hou, X.X.; Zheng, L.; Zhao, Y.P.; et al. Investigation of Lyme disease among residents of the Bogda Mountain forest area in Xilingol League, Inner Mongolia. *Chin. J. Zoonoses* **1996**, (1), 59. (In Chinese)

92. Lv, T.Z.; Feng, F.B.; Zhang, J.W.; Xie, M.; Zhou, G.P.; Tang, L. Epidemiological investigation of Lyme disease in a military unit stationed in a highly endemic area. *J. Prev. Med. Chin. PLA* **1997**, (4), 42–43. (In Chinese)

93. Ma, H.B.; Zhang, Q.; Yang, W.Y.; Pang, J.W. Report on Lyme disease antibody detection among febrile patients in Yingjiang County. *J. Dali Med. Coll.* **1994**, (3), 34–35, 58. (In Chinese)

94. Mi, J.X.; Wang, C.S.; Wan, K.L.; Yang, H.; Feng, Z.B.; Luo, J.; Zhang, G.F.; Liu, C.C. Epidemiological investigation and experimental study of Lyme disease. *Chin. J. Public Health Eng.* **2002**, (1), 47. (In Chinese)

95. Mo, Y.M.; Wang, G.Q.; Chen, X.Y.; Dong, C.H.; Zhu, G.Q.; Li, S.C.; Sun, R.L. Investigation of Lyme disease in Yingshan County, Hubei Province. *Chin. J. Public Health* **1994**, (3), 104–105. (In Chinese)

96. Ni, X.B.; Jia, N.; Jiang, B.G.; Sun, T.; Zheng, Y.C.; Huo, Q.B.; Liu, K.; Ma, L.; Zhao, Q.M.; Yang, H.; et al. Lyme borreliosis caused by diverse genospecies of *Borrelia burgdorferi* sensu lato in northeastern China. *Clin. Microbiol. Infect.* **2014**, 20(8), 808–814.

97. Pan, L.; Yu, E.S.; Lin, J.R.; Zhang, Z.F.; Cao, B.L.; Wang, Z.J.; Wu, S.S.; Liu, C.Z.; Zheng, Y.M.; Liu, J.Y.; et al. Discovery of Lyme disease in Fujian Province. *Chin. J. Zoonoses* **1990a**, (5), 63. (In Chinese)

98. Pan, L.; Yu, E.S.; Lin, J.R.; Zhang, Z.F.; Cao, B.L.; Wang, Z.J.; Wu, S.S.; Liu, C.Z.; Zheng, Y.M.; Liu, J.Y.; et al. Discovery of Lyme disease in Fujian Province. *Chin. J. Zoonoses* **1990b**, (5), 63. (In Chinese)

99. Qiu, H.Y.; Cao, H.; Qi, K. Investigation of *Borrelia burgdorferi* carriage in ticks near Songfeng Mountain. *Vet. Orient.* **2019**, (15), 76. (In Chinese)

100. Ren, G.S.; Yan, D.C.; Ran, X.L.; Zhang, B.C.; Han, M.; Liao, Y.Z. Seasonal dynamics of *Ixodes persulcatus* in a natural focus of Lyme disease in the Greater Khingan Mountains, Inner Mongolia. *J. Med. Pest Control* **1992**, (2), 107–109. (In Chinese)

101. Shen, J.J.; Zhang, J.S.; Jiang, W.M.; Pan, L.; Yu, E.S.; Tong, Y. Epidemiological status of Lyme disease along the northwestern border of Fujian Province. *Chin. J. Public Health* **1994**, (3), 166. (In Chinese)

102. Song, C.Y.; Jia, Y.H.; Chen, J.Y.; Liu, Y.; Yu, F.T. Lyme disease infection among primary and secondary school students in Jixian County, Tianjin. *Chin. J. Sch. Health* **2003**, (2), 181. (In Chinese)

103. Song, C.Y.; Liu, H.; Chen, J.Y.; Yu, F.T.; Zhao, Z.W.; Jia, Y.H.; Liu, Y. Investigation of the epidemiological status of Lyme disease in Jixian County, Tianjin. *Mod. Prev. Med.* **2012**, 39(20), 5419–5421. (In Chinese)

104. Song, X.L.; Zhang, J.T.; Lv, G.P.; Ge, M.X.; Han, F.M.; Zhang, Z.F.; Zhang, J.S.; Wang, H.Y.; Hou, X.X. Investigation of Lyme disease in the Sanchazi forest area of Hunjiang City. *Dis. Surveill.* **1994**, (4), 93–94. (In Chinese)

105. Sun, H.S.; Tian, Z.; Geng, Z.; Hou, X.X.; Hao, Q.; Jiang, Y.; Lv, B.; Wan, K.L. Epidemiological investigation of Lyme disease in the Xinjiang oil exploration area of the Henan Oilfield. *Chin. J. Vector Biol. Control* **2005**, (3), 209–211. (In Chinese)

106. Sun, X.; Zhang, G.L.; Zheng, Z.; Liu, X.M.; Liu, R.; Qiu, E.C.; Zhao, Y. Serological investigation of tick-borne diseases among forestry workers in northern Xinjiang. *Acta Parasitol. Med. Entomol. Sin.* **2016**, 23(3), 158–163. (In Chinese)

107. Tang, F.; Zhou, L.; Jiang, L.F.; Luan, J.; Fu, F.X. Investigation of *Borrelia burgdorferi* carried by vector ticks in the Qiqian region of Inner Mongolia. *Infect. Dis. Inf.* **2018**, 31(1), 31–33. (In Chinese)

108. Tang, K.; Zuo, S.Y.; Li, Y.; Zheng, Y.C.; Huo, Q.B.; Yu, J.H.; Zhang, Y.; Ni, X.B.; Yao, N.N.; Tan, H.Z. Dynamic investigation of coinfection with *Borrelia burgdorferi* and spotted fever group rickettsiae in ticks from tourist areas of Heilongjiang Province. *Chin. J. Epidemiol.* **2012**, 33(5), 513–516. (In Chinese)

109. Tan, Y.H.; Liu, Y.; Sun, H.; Yu, L.H.; Long, J.; Niu, X.S.; Mangunu; Mohetaer; Zhu, Y.; Wu, M. Surveillance of Lyme disease in the Xinjiang Uygur Autonomous Region from 2000 to 2004. *Chin. J. Clin. Neurosci.* **2007**, (2), 158–161. (In Chinese)

110. Tan, Y.H.; Liu, Y.; Sun, H.; Yu, L.H.; Mangunuer; Zhu, L.; Zhu, Y. Surveillance of a natural focus of Lyme disease in the southern mountains of Urumqi, Xinjiang. *Chin. J. Vector Biol. Control* **2011**, 22(2), 141–143. (In Chinese)

111. Tan, Y.H.; Liu, Y.; Wan, K.L.; Hao, Q.; Sun, H.; Yu, L.H.; Hou, X.X.; Geng, Z.; Zhang, Y.H.; Dang, H.; et al. Molecular epidemiological investigation of human infection with Lyme borreliae in Xinjiang. *Chin. J. Vector Biol. Control* **2013**, 24(4), 297–300. (In Chinese)

112. Tian, L.L.; Lv, Y.N.; Cao, D.Q.; Li, L.J.; Chen, Y.W.; Liu, X.F.; Li, S.; Dou, X.F.; Guan, Z.Z.; Wang, Q.Y.; et al. Investigation of Lyme disease infection among residents of Mentougou District, Beijing. *Acta Parasitol. Med. Entomol. Sin.* **2014**, 21(2), 92–96. (In Chinese)

113. Tian, M.F.; Han, Y.; Long, W.B.; Zhou, C.Q.; Wang, Z.X.; Lu, D.Q. Preliminary investigation of Lyme disease along the newly constructed Hengyang-Nanning railway line. *Railway Med. J.* **1997**, (1), 41. (In Chinese)

114. Tian, M.F.; Han, Y.; Zhu, J.L.; Wang, Z.X.; Luo, J.W. Serological survey of Lyme disease among outpatients and inpatients at Leiyang Railway Hospital, Hunan Province. *Pract. Prev. Med.* **1999a**, (6), 445. (In Chinese)

115. Tian, M.F.; Han, Y.; Long, W.B.; Zhou, R.Q.; Wang, Z.X.; Lu, D.Q. Serological survey of Lyme disease among railway construction workers. *Railway Med. J.* **1999b**, (5), 298–299. (In Chinese)

116. Tian, Z.; Wan, K.L.; Hou, X.X.; Hao, Q. Seroepidemiological survey of Lyme disease among populations in Xinjiang oil exploration areas. *Chin. J. Epidemiol.* **2003**, 24(8), 733. (In Chinese)
117. Wang, B.; Ma, C.P.; Zhu, W.F.; Wang, F.L. Seroepidemiological survey of Lyme disease and associated factors in selected areas of Gansu Province. *Med. J. Natl. Defending Forces Southwest China* **2020**, 30(10), 971–972. (In Chinese)
118. Wang, C.S.; Du, Z.S.; Yang, X.J.; Yang, H.; Wan, K.L.; Yang, X.M.; Liu, W.Y.; Guo, Y.X.; Zhang, X.W.; Dai, X.X. First identification of a Lyme disease focus in the Changchun region. *Chin. J. Public Health Eng.* **2005**, (3), 158–160. (In Chinese)
119. Wang, C.S.; Wan, K.L.; Guo, J.H.; Geng, Z.; Wang, B.; Chen, D.G.; Zhao, Z.F.; Xia, Q.B.; Yang, H. Epidemiological status of Lyme disease in border areas of Jilin Province. *J. Public Health Prev. Med.* **2008**, (5), 48–49. (In Chinese)
120. Wang, C.S.; Wan, K.L.; Yang, X.J.; Wang, B.; Zhang, G.F.; Liang, X.P.; Guo, J.H.; Hou, Y.M.; Yang, H. Epidemiological investigation of Lyme disease in the plains of Jilin Province. *Pract. Prev. Med.* **2009a**, 16(2), 341–342. (In Chinese)
121. Wang, C.S.; Wan, K.L.; Yang, X.J.; Zhang, G.F.; Liu, F.C.; Shi, D.W.; Dong, C.; Wang, P. Investigation of Lyme disease distribution in Yanbian and Jilin. *Pract. Prev. Med.* **2009b**, 16(4), 1074–1075. (In Chinese)
122. Wang, G.Q.; Mo, Y.M.; Chen, X.Y.; Dong, C.H.; Zhu, G.Q.; Sun, R.L.; Li, S.C. Discovery of Lyme disease in northeastern Hubei Province. *J. Hubei Med. Univ.* **1994**, (4), 332–335. (In Chinese)
123. Wang, H.W.; He, J.; Chu, C.Y.; Zhang, P.H.; Wu, X.M.; Huang, H.N.; Gao, Y.; Jiang, B.G.; Cao, W.C. Detection and genotyping of Lyme borreliae in ticks from selected regions of China. *J. Pathog. Biol.* **2006**, 1(2), 81–85. (In Chinese)
124. Wang, J.J.; Fang, H.L.; Huang, H.; Duan, X.M.; Tang, W.J.; Tan, B. Epidemiological analysis of myocardial injury associated with Lyme disease. *Clin. Focus* **2010**, 25(9), 746. (In Chinese)
125. Wang, J.Z.; Yuan, P.; Guo, H.L.; Yang, J.L.; Xu, Z.G.; Liu, Z.J. Preliminary epidemiological investigation of Lyme disease among residents of Shiyan City. *J. Yunyang Med. Coll.* **2004**, (6), 370. (In Chinese)
126. Wang, L.J.; Tao, X.R.; Jiang, J.T.; Ji, Q.T.; Wei, Q.L.; Chen, Z.L.; Zhang, Z.F.; Wang, H.Y.; Zheng, L.; Wan, K.L. Investigation of Lyme disease in Shandong Province. *Chin. J. Vector Biol. Control* **1992**, (4), 229–231. (In Chinese)
127. Wang, L.J.; Wan, K.L.; Liu, S.L.; Chen, Z.L.; Feng, K.J.; Hou, X.X.; Shi, C.X. First investigation demonstrating the presence of natural foci of Lyme disease in Shandong Province. *Chin. J. Epidemiol.* **2000**, 21(4), 292–294. (In Chinese)
128. Wang, L.J.; Hou, X.X.; Chen, Z.L.; Hao, Q.; Tao, X.R.; Wan, K.L. Geoepidemiological study of Lyme disease in Shandong Province. *Chin. J. Vector Biol. Control* **2007**, (4), 306–308. (In Chinese)
129. Wang, R.K.; Sun, H.Y. Investigation report on Lyme disease in Langxi County. *Anhui Med. J.* **1990**, (6), 40–41. (In Chinese)
130. Wang, S.Q.; Zhan, B.D.; Cao, G.P.; Yu, Z.Y.; Zhang, J.M. *Borrelia burgdorferi* infection among older adults and indoor rodents in Kecheng District. *Prev. Med.* **2018**, 30(12), 1252–1254. (In Chinese)
131. Wang, X.L.; Yang, X.J.; Guo, J.H.; Wang, C.S. Distribution and clinical diagnosis of Lyme disease in the Changchun region. *Chin. J. Health Lab. Technol.* **2013**, 23(9), 2160–2161. (In Chinese)
132. Wang, Z.J.; Tang, J.H.; Lai, H.N.; Li, D.M.; Xu, Z.X. Seroepidemiological survey of Lyme disease in the forest areas of northern Fujian. *Chin. J. Public Health* **1991**, (1), 49. (In Chinese)

133. Wang, Z.X.; Wang, D.M.; Weng, C.R.; Tang, G.P.; Liu, M.; Hu, L.J.; Li, Z.Y.; Cai, X.H.; Huang, Y.P.; Fan, J.G. Investigation of Lyme disease in Guizhou Province. *Chin. J. Zoonoses* **2003**, (2), 113–114. (In Chinese)
134. Wen, S.; Xu, Q.; Liu, D.; Lin, Z.; Lin, Z.; Chen, S.; Chen, M. A seroepidemiological investigation of Lyme disease in Qiongzhong County, Hainan Province in 2019-2020. *Ann. Palliat. Med.* **2021**, 10(4), 4721–4727.
135. Wu, B.X.; Lin, P.; Wu, Q.L.; Tong, Y.; Pan, L. Investigation and prevention measures for Lyme disease in forest areas of Fujian Province. *Pract. Prev. Med.* **1994**, 1(3), 137–138. (In Chinese)
136. Wu, J.Y.; Gong, T.M.; Wang, L.; Jiang, W.; Ke, Y. Seroepidemiological survey of tick-borne Lyme disease among different populations in Kashgar, Xinjiang. *J. Trop. Med.* **2017**, 17(12), 1663–1665, 1673. (In Chinese)
137. Wu, S.S.; Pan, L.; Lin, J.X.; Cai, H.; Ceng, Y.S.; Wu, L.F.; Zhu, Y.S. Evidence of Lyme disease infection in Jian'ou County. *Chin. J. Public Health* **1991**, (2), 80. (In Chinese)
138. Wu, Y.M.; Wei, A.M.; Liu, X.X.; Wang, G.; Hu, L.M.; Yang, Q. Serological survey of three tick-borne diseases in the Suifenhe region. *J. Prev. Med. Chin. PLA* **1997**, (5), 34–36. (In Chinese)
139. Xiao, G.L.; Yan, X.L.; Liu, G.Z.; Sheng, S.D.; Xu, P.X.; Shi, R.B.; Cai, B.C. Report of a seroepidemiological survey of Lyme disease in Jiangsu Province. *Chin. J. Vector Biol. Control* **1992**, (2), 104–106. (In Chinese)
140. Xia, Q.B.; Wang, C.S.; Li, Y.X.; Dai, J.L.; Chen, D.G.; Ge, Y.H.; Du, Y.J.; Liang, G.S. Investigation and analysis of Lyme disease in the Tonghua region. *Chin. J. Health Lab. Technol.* **2007**, (10), 1857–1858. (In Chinese)
141. Xie, X.C.; Liu, H.J.; Ye, E.K.; Yang, X.K.; Abulikemu; Wang, C.; Wushoubate; Silamujiang. Geographical distribution and epidemiological status of Lyme disease in Xinjiang. *Endem. Dis. Bull.* **1996**, 11(3), 48–50. (In Chinese)
142. Xing, F.K.; Peng, Z.H.; Gao, G.H.; Zhang, J.; Wang, L.X.; Hou, X.X.; Wan, K.L. Preliminary investigation of Lyme disease in Changping District, Beijing. *Chin. J. Zoonoses* **2002**, (6), 106–110. (In Chinese)
143. Xu, X. Current status and prevention of Lyme disease infection among workers from the Henan Oilfield pilot-production company deployed to Xinjiang. *Henan J. Prev. Med.* **2000**, (3), 168. (In Chinese)
144. Yan, D.C.; Zhang, Z.F.; Wang, J.B.; Zhang, J.S.; Zhu, J.H.; Wang, H.Y.; Liao, Y.Z.; Cao, B.L.; Zhang, B.C.; Wang, S.Y.; et al. *Ixodes persulcatus* as the principal biological vector of Lyme borreliae in the Greater Khingan Mountains of Inner Mongolia. *Chin. J. Vector Biol. Control* **1991**, (4), 270–272. (In Chinese)
145. Yang, J.C. Seroepidemiological survey of Lyme disease in Xuzhou City. *Chin. J. Public Health* **2001**, (4), 60. (In Chinese)
146. Yang, L.; Zhang, Z.Y.; Shao, T.T.; Li, X.T.; Zheng, R.J.; Wang, S.Y.; Wang, Y.Z.; Hao, Q.; Lu, X.B. Seroepidemiological survey of Lyme disease in Urumqi, Xinjiang. *Chin. J. Zoonoses* **2024**, 40(4), 334–339. (In Chinese)
147. Yang, X.F.; Zhang, Z.F.; Lu, J.P.; Zhang, J.S.; Zheng, L.; Hou, X.X.; Chen, B.; Chen, Y.S.; Dong, J.J.; Zhang, Z.H.; et al. Investigation of Lyme disease in the southern Greater Khingan Mountains. *Chin. J. Vector Biol. Control* **1992**, (1), 39–41. (In Chinese)
148. Yang, X.F.; Lu, J.P.; Zhang, J.L.; Fan, Y.L.; Yuan, J. Investigation of Lyme disease infection among selected populations in Baotou and Hohhot. *Chin. J. Vector Biol. Control* **1996**, (1), 41. (In Chinese)
149. Yang, X.F.; Cheng, K.L.; Lu, J.P.; Temuqihu; Zhang, Z.F.; Han, X.Z.; Zhao, G.; Zhang, X.S.; Li, M.Q. Geoepidemiological and etiological investigation of Lyme disease in Inner Mongolia. *Chin. J. Zoonoses* **1999**, 15(2), 98. (In Chinese)
150. Yang, X.J.; Wang, C.S.; Gao, L.Y.; Yang, H.; Ge, Y.H.; Liu, W.X.; Quan, Y.W.; Liu, D.G.; Liu, F.C.; Liu, Y.Y.; et al. Investigation of Lyme disease in the Changbai Mountain area. *Chin. J. Zoonoses* **2001a**, 17(2), 104. (In Chinese)

151. Yang, X.J.; Wang, C.S.; Wan, K.L.; Shi, D.P.; Ge, Y.H.; Liu, W.X.; Quan, Y.W.; Liu, D.G.; Liu, F.C.; Liu, Y.Y.; et al. Investigation and laboratory analysis of Lyme disease in the Changbai Mountain area. *Chin. J. Vector Biol. Control* **2001b**, (2), 115–117. (In Chinese)

152. Yang, X.J.; Wan, K.L.; Wang, C.S.; Geng, Z.; Guo, J.H.; Wang, B.; Wang, H.; Zhang, G.F.; Gu, Y. Detection of Lyme disease in human populations using the indirect fluorescent antibody assay. *Chin. J. Health Lab. Technol.* **2009**, 19(2), 345–346. (In Chinese)

153. Yang, Y.S.; Lv, Y.N.; Chen, Y.L. Serological survey of Lyme disease among patients with arthritis in Miyun District, Beijing. *Chin. J. Vector Biol. Control* **2017**, 28(5), 490–491. (In Chinese)

154. Yan, R.J.; Quan, Y.W.; Ji, W.X. Epidemiological investigation of Lyme disease among residents of forest areas in Huadian City. *Pract. Prev. Med.* **1995**, (2), 81. (In Chinese)

155. Yan, X.L. Seroepidemiological survey of Lyme disease in Jiangsu Province. *Chin. J. Public Health* **2000**, 16(1), 38. (In Chinese)

156. Yan, X.L. Seroepidemiology of Lyme disease pathogens and their distribution in Jiangsu Province. *Chin. J. Sch. Dr.* **2003**, (1), 29–30. (In Chinese)

157. Ye, J.C.; Xu, L.C.; Gao, Y.P.; Tian, X.K.; Han, M.H.; He, X.; Shen, Z.; Wang, J.; Zhang, F.Q. Investigation of Lyme disease in Mentougou District, Beijing. *Dis. Surveill.* **1994**, (7), 173–175. (In Chinese)

158. Ye, X.; Wang, R.F.; Li, G.W. Investigation of Lyme disease infection and tick vectors among selected populations in Xiamen. *J. Prev. Med. Inf.* **2007**, (3), 282–283. (In Chinese)

159. Yin, F.R. Investigation of Lyme borrelia infection among psychiatric patients in the Greater Khingan forest area. *Chin. J. Public Health Manag.* **2008**, (2), 212–213. (In Chinese)

160. Yu, D.S.; Geng, Z.; Jiang, J.X.; Hao, Q.; Chen, J.H.; Wang, P. Investigation of a natural focus of Lyme disease in Diebu County, Gansu Province. *Chin. J. Vector Biol. Control* **2009**, 20(1), 57–58. (In Chinese)

161. Yue, J.N.; Shi, Y. Epidemiological investigation of Lyme disease in selected forest areas of Qinghai Province. *Chin. J. Vector Biol. Control* **2009**, 20(4), 358–359. (In Chinese)

162. Zhang, D.R.; Hang, H.G.; Liu, H.; Gu, L.L.; Yu, Z.C.; Luo, Z.Z. Seroepidemiological survey of Lyme disease among residents of Anhui Province. *Anhui J. Prev. Med.* **1996**, (1), 27–29. (In Chinese)

163. Zhang, D.R.; Hang, H.G.; Li, Q. Preliminary investigation of Lyme disease infection among residents of Nyingchi, Tibet. *Chin. J. Zoonoses* **1997a**, (2), 70. (In Chinese)

164. Zhang, D.R.; Lin, T.; Li, Q.; Wang, J.J.; Zhang, M.J.; Zhang, Z.F.; Xue, Z.Y.; Hu, S.S. Seroepidemiological study of Lyme disease in Anhui Province. *Chin. J. Vector Biol. Control* **1998**, (4), 33–35. (In Chinese)

165. Zhang, F.Z.; Liang, W.N. A newly recognized natural-focal disease in Anhui Province: Lyme disease. *Acta Univ. Med. Anhui* **1989**, (3), 180–185. (In Chinese)

166. Zhang, J.J.; Liu, Z.J.; Zhang, F.; Gong, Z.W.; Liu, H. Molecular epidemiological investigation of Lyme borreliae in the Altay region. *Acta Parasitol. Med. Entomol. Sin.* **2011**, 18(1), 34–37. (In Chinese)

167. Zhang, J.M.; Yang, H.J.; Li, X.M.; Gao, X.F.; Yang, T.; Zhao, Z.S.; Zhao, C.X.; Zhao, L.H.; Geng, Z.; Wan, K.L. Investigation of Lyme disease infection in a county of Shanxi Province. *Chin. Remedies Clin.* **2006**, (7), 490–491. (In Chinese)

168. Zhang, L.; Zhu, X.; Hou, X.; Li, H.; Yang, X.; Chen, T.; Fu, X.; Miao, G.; Hao, Q.; Li, S. Prevalence and prediction of Lyme disease in Hainan province. *PLoS Negl. Trop. Dis.* **2021**, 15(3), e0009158.

169. Zhang, L.B.; Cheng, J.F.; Wan, K.L.; Hou, X.X.; Lin, F.R.; Zhang, Y.; Wu, H.X.; Xiang, X.S.; Hu, Z.Y.; Lin, Z.Q.; et al. Seroepidemiological study of Lyme disease in Hubei Province. *Chin. J. Vector Biol. Control* **1999**, 10(3), 176–177. (In Chinese)
170. Zhang, L.L.; Hou, X.X.; Geng, Z.; Huo, Q.B.; Hao, Q.; Wan, K.L.; Lou, Y.L. Application of nested PCR to the detection of serum samples from patients with suspected Lyme disease. *Chin. J. Vector Biol. Control* **2013**, 24(1), 8–10. (In Chinese)
171. Zhang, L.L.; Hou, X.X.; Geng, Z.; Lou, Y.L.; Wan, K.L.; Hao, Q. Combination of Loop-Mediated Isothermal Amplification Assay and Nested PCR for Detection of *Borrelia burgdorferi* sensu lato in Human Serum Samples. *Biomed. Environ. Sci.* **2015**, 28(4), 312–315.
172. Zhang, P.H.; Chen, W.R.; Wu, Z.C.; Zhang, Q.E. Establishment and preliminary application of a competitive McAb-ELISA for detection of Lyme disease. *J. Cell. Mol. Immunol.* **1992**, (2), 35–38. (In Chinese)
173. Zhang, Q.Q.; Niu, J.Q.; Yang, Q.F.; Liu, L.M.; Guan, X.C. Investigation report on Lyme disease in the Changbai Mountain forest area of Jilin Province. *Chin. J. Epidemiol.* **1996**, (2), 116. (In Chinese)
174. Zhang, X.; Zheng, X.Y.; Zhao, X. Investigation report on Lyme disease in Hebei Province. *J. Prev. Med. Chin. PLA* **1993**, (5), 365–366. (In Chinese)
175. Zhang, X.C.; Yang, Z.N.; Lu, B.; Ma, X.F.; Zhang, C.X.; Xu, H.J. The composition and transmission of microbiome in hard tick, *Ixodes persulcatus*, during blood meal. *Ticks Tick-Borne Dis.* **2014**, 5(6), 864–870.
176. Zhang, X.P.; Wang, D.H.; Su, D.S.; Luo, Y.Q. Investigation of Lyme disease in the Huajian area. *Chin. J. Vet. Sci. Technol.* **1994**, (10), 15–16. (In Chinese)
177. Zhang, X.P.; Liu, Z.J.; Shi, S.Z.; Zhang, J.; Yang, Y.S.; Wang, D.H. Serological survey of Lyme disease among residents of Diebu at the northern foot of the Minshan Mountains, Gansu Province. *Chin. J. Zoonoses* **1997b**, (4), 72–73. (In Chinese)
178. Zhang, Z.D.; Chen, Y.J.; Mei, J.H.; Wang, D.B.; Lin, R.W.; Wu, W.Y.; Lv, L.Z.; Wang, C.; Shi, G.P. Seroepidemiological study of Lyme disease among residents of southern Zhejiang Province. *Chin. J. Public Health* **2001**, (11), 79–80. (In Chinese)
179. Zhang, Z.K.; Tian, W.C.; Guo, Y.X.; Li, Y.P.; Zhang, Z.F.; Zhang, J.S. Seroepidemiological survey of Lyme disease among selected populations in Hebei Province. *Chin. J. Public Health* **1993**, (5), 240. (In Chinese)
180. Zhao, Q.M.; Wu, X.M.; Zhang, P.H.; Li, J.M.; Yang, H.; Wei, M.T.; Zhang, X.T.; Cao, W.C. Study of coinfection with three tick-borne diseases in vector ticks and rodents. *Chin. J. Epidemiol.* **2005**, (1), 12–16. (In Chinese)
181. Zhao, Y.; Liu, R.; Zhang, G.L.; Liu, X.M.; Sun, X.; Zheng, Z.; Qiu, E.C. Seroepidemiological survey of tick-borne diseases in a border-defense unit in northern Xinjiang. *J. Prev. Med. Chin. PLA* **2014**, 32(4), 324–325. (In Chinese)
182. Zhou, Q.Y.; Zhao, M.Y.; Huang, Z.Y.; Liu, L.S.; Wang, W.S. First detection of Lyme disease antibodies in human sera from Sichuan Province. *J. Prev. Med. Inf.* **1990**, (3), 134. (In Chinese)
183. Zhou, Q.Y.; Zhao, M.Y.; Huang, Z.Y.; Liu, L.S.; Wang, W.S.; Wan, K.L. Seroepidemiological survey of Lyme disease among healthy residents of Sichuan Province. *J. Prev. Med. Inf.* **1991**, (1), 46–47, 38. (In Chinese)
184. Zhuang, Y.; Wang, D.M.; Jiang, W.J.; Zhou, J.Z.; Hu, J.; Yu, C. Investigation of Lyme disease infection among rural residents of Guizhou Province in 2006. *Guizhou Med. J.* **2009**, 33(2), 169–170. (In Chinese)

185. Zhu, X.; Zhang, L.; Hou, X.X.; Geng, Z.; Chen, H.; Chen, T.; Yu, S.Y.; Hao, Q. Investigation of Lyme disease among patients with arthritis and neurological disorders in Hainan Province. *Chin. J. Zoonoses* **2015**, 31(4), 353–356. (In Chinese)

186. Zhu, X.; Hou, X.X.; Yu, L.; Zhang, L.; Chen, Y.Y.; Miao, G.Q.; Chen, T.; Hao, Q.; He, L.F. Serological survey of Lyme disease in western Hainan Province. *Chin. J. Zoonoses* **2020**, 36(4), 313–316, 319. (In Chinese)

Supplementary File S3

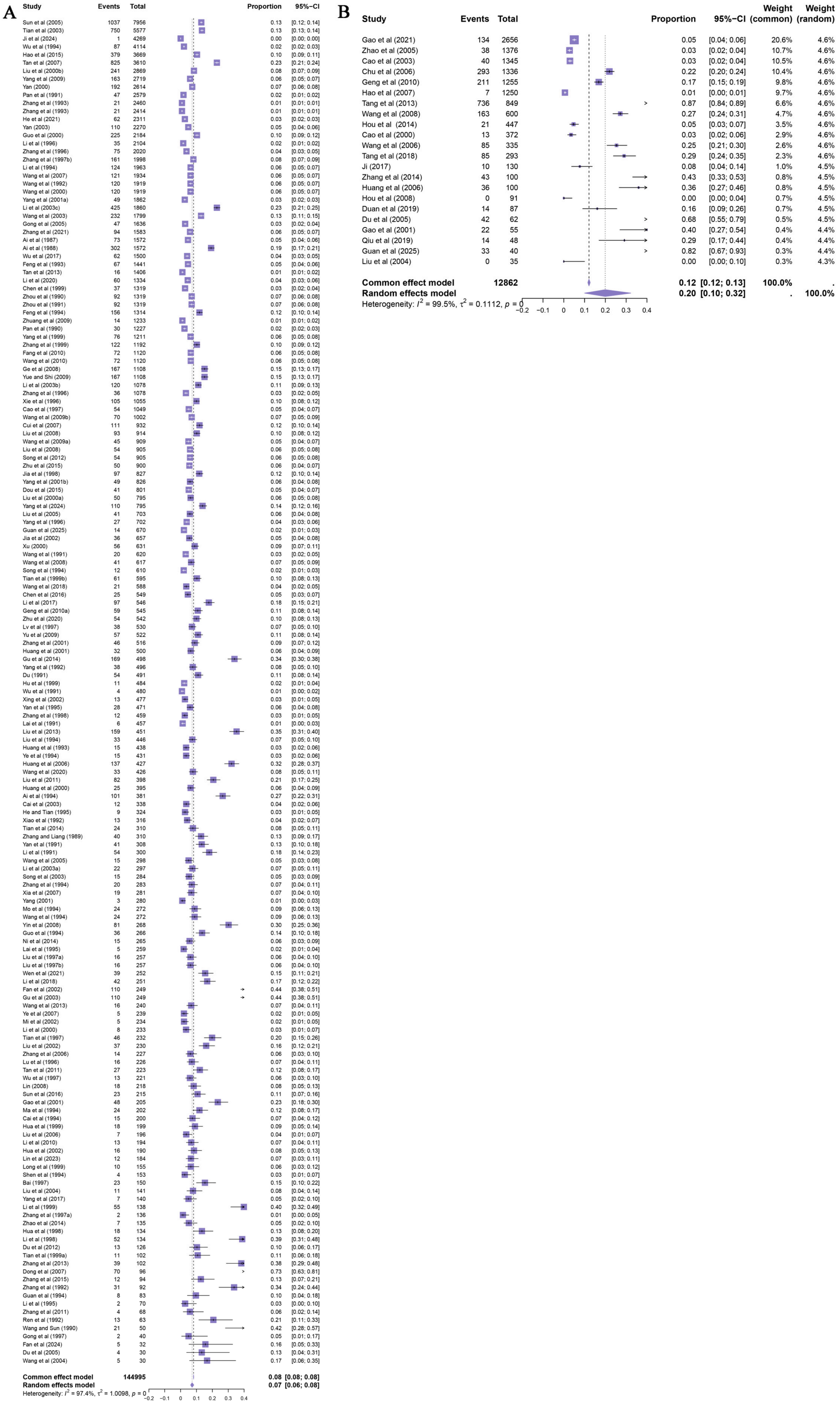

Figure S1. Forest plots of *Borrelia burgdorferi* sensu lato prevalence in humans and questing or unfed *Ixodes* ticks in China. (A) Random-effects meta-analysis of human seroprevalence, including 167 studies comprising 144,995 examined individuals and 11,867 seropositive individuals. The pooled seroprevalence was 7% (95% CI: 6%–8%), with substantial between-study heterogeneity (I² = 97.4%). (B) Random-effects meta-analysis of PCR-confirmed infection prevalence in questing or unfed *Ixodes* ticks, including 22 studies comprising 12,862 examined ticks and 2,040 PCR-positive ticks. The pooled infection prevalence was 20% (95% CI: 10%–32%), with substantial between-study heterogeneity (I² = 99.5%). Individual study estimates and the corresponding pooled estimates are presented with 95% confidence intervals.

microorganisms

Supplementary File S4

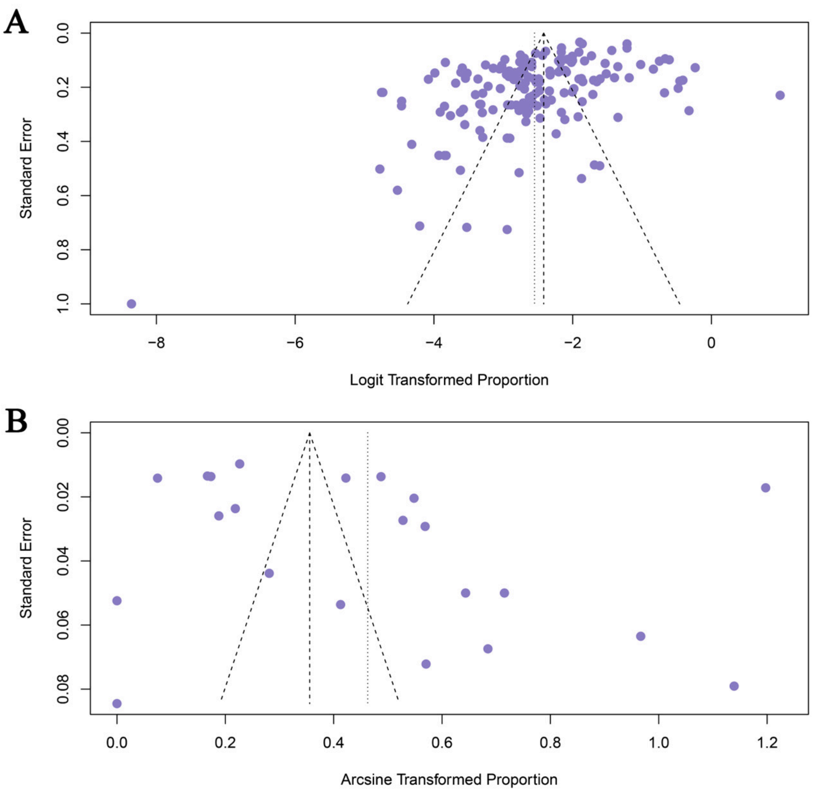

Figure S2. Funnel plots of *Borrelia burgdorferi* sensu lato seroprevalence in humans and infection prevalence in questing Ixodes ticks in China. Panels A and B show the original funnel plots for human seroprevalence and infection prevalence in questing Ixodes ticks, respectively. Filled circles represent the observed studies. Standard errors are plotted against logit-transformed proportions for human studies (A) and arcsine-transformed proportions for tick studies (B). The vertical reference lines indicate the pooled effect estimates, and the diagonal dashed lines represent the pseudo 95% confidence limits.

Supplementary File S5

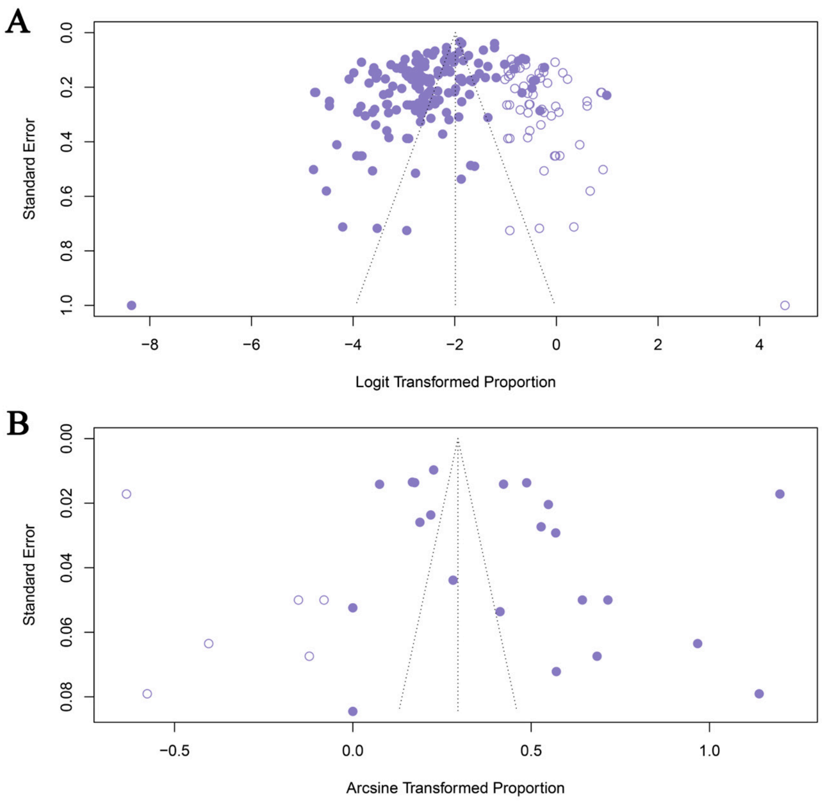

Figure S3. Trim-and-fill-adjusted funnel plots of *Borrelia burgdorferi* sensu lato seroprevalence in humans and infection prevalence in questing Ixodes ticks in China. Panels A and B show the adjusted funnel plots for human seroprevalence and infection prevalence in questing Ixodes ticks, respectively. Filled circles represent the observed studies, whereas open circles represent potentially missing studies imputed using the trim-and-fill method. Standard errors are plotted against logit-transformed proportions for human studies (A) and arcsine-transformed proportions for tick studies (B). The vertical reference lines indicate the pooled effect estimates after trim-and-fill adjustment, and the diagonal dotted lines represent the pseudo 95% confidence limits.

Supplementary File S6

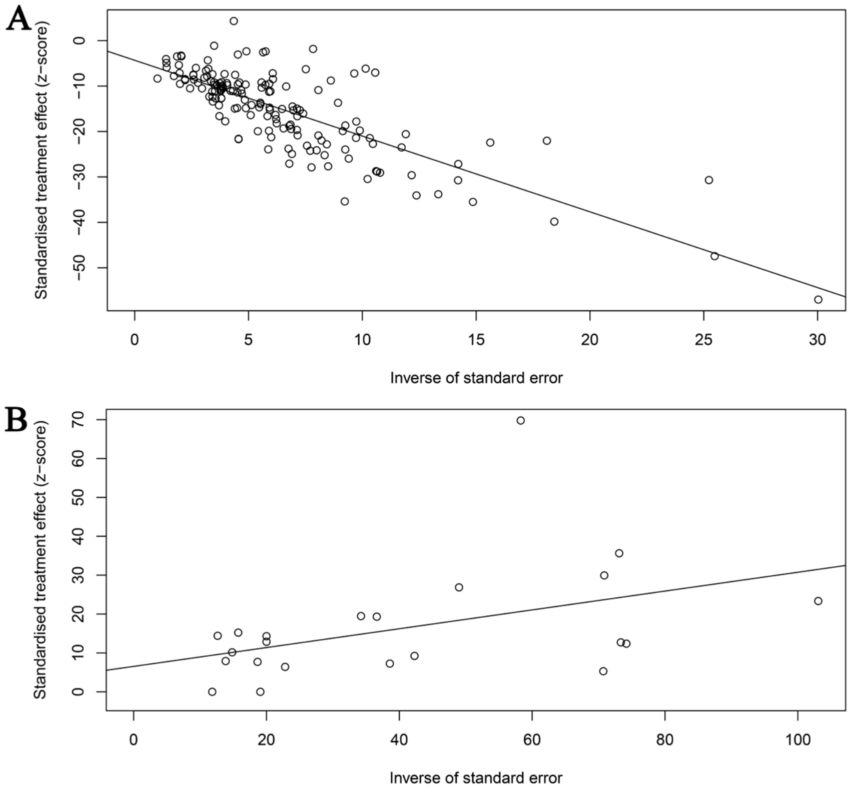

Figure S4. Egger's regression plots assessing small-study effects in studies of Borrelia burgdorferi sensu lato prevalence in China. Panel A shows human seroprevalence studies, and panel B shows infection-prevalence studies of questing Ixodes ticks. Each open circle represents an individual study, with the standardized effect estimate (z score) plotted against the inverse of its standard error; the solid line represents the fitted Egger regression line. Significant funnel-plot asymmetry was detected for human studies ($t = -5.40$, $df = 165$, $P < 0.0001$; bias estimate = −4.3313, SE = 0.8026), indicating evidence of small-study effects. In contrast, no significant funnel-plot asymmetry was detected for tick studies ($t = 1.20$, $df = 20$, $P = 0.2431$; bias estimate = 6.5355, SE = 5.4338).

Supplementary File S7

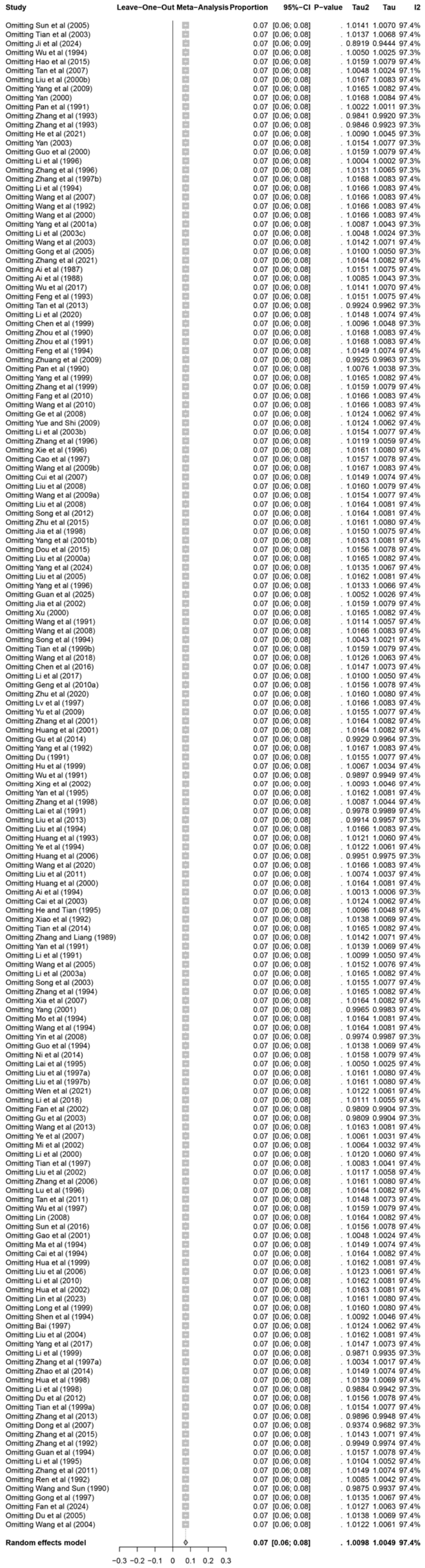

Figure S5. Leave-one-out sensitivity analysis of the seroprevalence of Borrelia burgdorferi sensu lato in humans in China. Each row presents the pooled seroprevalence and corresponding 95% confidence interval obtained from the random-effects model after sequential omission of one study. The pooled estimates remained approximately 7% (95% CI: 6%–8%) across all iterations, indicating that no individual study materially influenced the overall estimate. The diamond at the bottom represents the pooled estimate based on all included human studies. Substantial residual heterogeneity remained (I^2 = 97.4%).

Supplementary File S8

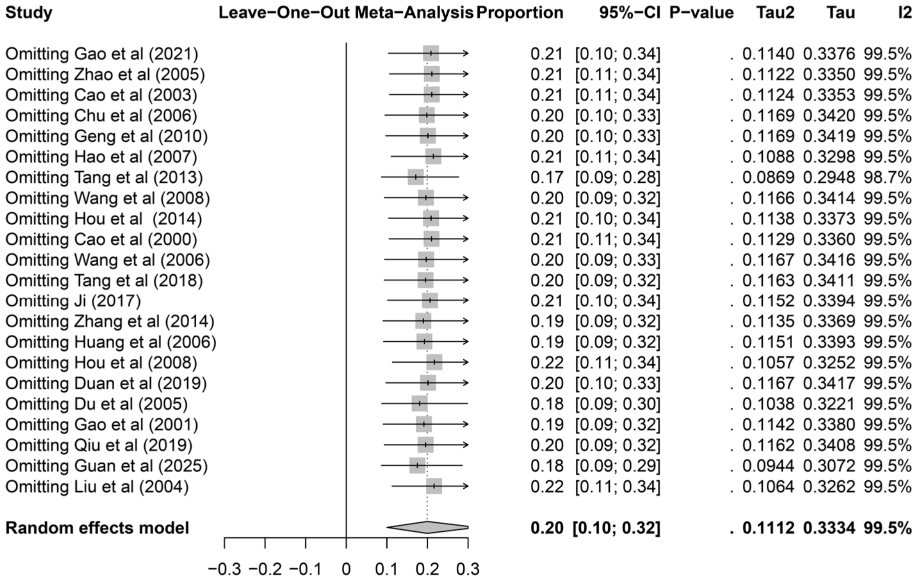

Figure S6. Leave-one-out sensitivity analysis of the infection prevalence of Borrelia burgdorferi sensu lato in questing Ixodes ticks in China. Each row presents the pooled infection prevalence and corresponding 95% confidence interval obtained from the random-effects model after sequential omission of one study. The pooled estimates remained close to 20% (95% CI: 10%–32%) across all iterations, indicating that the overall result was not disproportionately influenced by any single study. The diamond at the bottom represents the pooled estimate based on all included tick studies. Substantial residual heterogeneity remained ($I^2 = 99.4\%$).

microorganisms

Supplementary File S9

Table S2. Summary of pooled human seroprevalence of *Borrelia burgdorferi* sensu lato exposure and PCR-confirmed infection prevalence in questing *Ixodes* ticks in China.

	No. stud-ies	No. examined	No. positive	% (95% Cl)	Heterogeneity			Univariate meta-regression	
					χ2	*p*-value	I² (%)	*p*-value	Coefficient (95% CI)
**Humans**									
**Geographical region**									
Central China	19	18366	1921	0.0925 [0.0773-0.1088]	213.23	$p < 0.0001$	91.6	0.1855	-0.0521 (-0.1293 to 0.0250)
Eastern China	29	29851	1452	0.0510 [0.0355-0.0691]	549.15	$p < 0.0001$	94.9	<.0001	-0.1347 (-0.2024 to -0.0669)
Northeastern China	35	27548	2230	0.1310 [0.0838-0.1870]	2901.52	$p = 0$	98.8	-	-
Northern China	27	19392	1422	0.0798 [0.0554-0.1082]	1271.42	$p < 0.0001$	98.0	0.0298	-0.0771 (-0.1466 to -0.0076)
Northwestern China	31	26959	3115	0.0900 [0.0725-0.1092]	1107.64	$p < 0.0001$	97.3	0.0766	-0.0603 (-0.1271 to 0.0064)
Southwestern China	15	11395	788	0.0540 [0.0318-0.0815]	414.14	$p < 0.0001$	96.6	0.0029	-0.1284 (-0.2130 to -0.0439)
Western China	7	7173	401	0.0784 [0.0445-0.1208]	128.34	$p < 0.0001$	95.3	0.1637	-0.0787 (-0.1895 to 0.0321)
**Sampling year**									
1986-1995	49	46246	2696	0.0637 [0.0494-0.0797]	1723.65	$p = 0$	97.2	0.0263	-0.0629 (-0.1184 to -0.0074)
1996-2005	37	23327	2669	0.0983 [0.0724-0.1277]	1877.85	$p = 0$	98.1	-	-
2006-2015	23	14273	866	0.0863 [0.0547-0.1241]	425.56	$p < 0.0001$	94.8	0.4969	-0.0237 (-0.0919 to 0.0446)
2016-2025	8	8236	278	0.0556 [0.0222-0.1030]	591.77	$p < 0.0001$	98.8	0.1094	-0.0807 (-0.1795 to 0.0181)
**Age (years)**									
<20	27	6338	435	0.0673 [0.0506-0.0862]	176.11	$p < 0.0001$	85.2	0.0368	-0.0621 (-0.1204 to -0.0038)
>50	34	6878	666	0.0892 [0.0686-0.1122]	243.56	$p < 0.0001$	86.5	0.4408	-0.0216 (-0.0765 to 0.0333)
20-30	31	8984	799	0.0826 [0.0639-0.1034]	283.19	$p < 0.0001$	89.4	0.2506	-0.0327 (-0.0884 to 0.0231)
30-40	32	13033	1564	0.1019 [0.0772-0.1296]	568.22	$p < 0.0001$	94.5	-	-

**40-50**	**34**	**10317**	**1074**	**0.0936 [0.0706-0.1195]**	**427.92**	***p* < 0.0001**	**92.3**	**0.6087**	**-0.0143 (-0.0690 to 0.0404)**
Sex									
Man	63	27353	2137	0.0858 [0.0697-0.1033]	1165.31	*p* < 0.0001	94.7	-	-
Women	60	21598	1618	0.0797 [0.0632-0.0979]	1014.86	*p* < 0.0001	94.2	0.6168	-0.0112 (-0.0551 to 0.0327)
Season									
Spring	9	4019	234	0.0551 [0.0407-0.0714]	35.17	*p* < 0.0001	77.3	0.0735	-0.1869 (-0.3916 to 0.0177)
Winter	2	1550	83	0.1892 [0.0000-0.6524]	48.45	*p* < 0.0001	97.9	-	-
Autumn	7	5300	371	0.0633 [0.0184-0.1325]	255.52	*p* < 0.0001	97.7	0.1163	-0.1677 (-0.3769 to 0.0416)
Summer	18	11869	737	0.0714 [0.0450-0.1034]	850.40	*p* < 0.0001	98.0	0.1302	-0.1514 (-0.3474 to 0.0447)
**Tick bites**									
**Not suffering**	**12**	**10159**	**406**	**0.0506 [0.0229-0.0885]**	**416.93**	***p* < 0.0001**	**97.4**	**-**	**-**
**Suffering**	**99**	**94255**	**8564**	**0.1025 [0.0837-0.1230]**	**5170.94**	***p* =0**	**98.1**	**0.0465**	**0.0987 (0.0015 to 0.1959)**
**Risk**									
**Low**	**19**	**9857**	**1058**	**0.0883 [0.0498-0.1365]**	**1028.30**	***p* < 0.0001**	**98.2**	**0.6350**	**-0.0177 (-0.0908 to 0.0554)**
**High**	**87**	**84790**	**7406**	**0.0984 [0.0808-0.1177]**	**3553.32**	***p* = 0**	**97.6**	**-**	**-**
**Moderate**	**13**	**9384**	**266**	**0.0489 [0.0299-0.0724]**	**235.72**	***p* < 0.0001**	**94.9**	**0.0466**	**-0.0882 (-0.1751 to -0.0013)**
Residence									
Urban	16	8129	860	0.0706 [0.0404-0.1085]	672.79	*p* < 0.0001	97.8	0.3758	-0.0372 (-0.1195 to 0.0451)
Rural	103	92714	7485	0.0908 [0.0739-0.1093]	4776.31	*p* = 0	97.9	-	-
Antibodies									
IgG	100	69926	5610	0.0883 [0.0724-0.1056]	3203.57	*p* = 0	96.9	-	-
IgG + IgM	18	24604	2575	0.0860 [0.0570-0.1203]	884.55	*p* < 0.0001	98.1	0.9205	-0.0037 (-0.0759 to 0.0685)
IgM	5	7471	708	0.0661 [0.0219-0.1320]	467.85	*p* < 0.0001	99.1	0.5289	-0.0413 (-0.1701 to 0.0874)
Pathogen species									
B.afzelii	3	1215	86	0.0754 [0.0512-0.1039]	5.36	*p* = 0.0687	62.7	0.7657	-0.0444 (-0.3364 to 0.2476)
B.garinii	2	1087	113	0.1039 [0.0865-0.1227]	0.22	*p* = 0.6412	0.0	-	-
B.garinii + *B.afzelii*	3	2835	223	0.1019 [0.0016-0.3286]	326.69	*p* < 0.0001	99.4	0.9782	-0.0041 (0.9782 to 0.2870)
Detection Method									
ELISA	22	11705	1049	0.0828 [0.0569-0.1189]	936.28	*p* < 0.0001	97.8	0.8700	-0.1219 (-1.2518 to 1.3380)
ELISA+WB	1	310	24	0.0774 [0.0524-0.1129]	0.00	-	-	0.8646	-0.2103 (-2.6268 to 2.2063)

IFA	121	103621	8314	0.0750 [0.0632-0.0889]	4258.99	*p* = 0	97.2	0.7537	-0.2256 (-1.6349 to 1.1836)
IFA+ELISA	2	8111	1047	0.1291 [0.1220-0.1366]	5.58	*p* = 0.0182	82.1	-	-
IFA+WB	2	765	81	0.1059 [0.0860-0.1297]	0.77	*p* = 0.3817	0.0	0.8567	0.1812 (-1.7585 to 2.1481)
WB	6	3996	255	0.0614 [0.0253-0.1414]	171.13	*p* < 0.0001	97.1	0.5952	-0.4373 (-2.0501 to 1.1756)
**Ticks**									
**Geographical region**									
**NORTHEASTERN CHINA**	**12**	**3999**	**1203**	**0.2941 [0.1497-0.4637]**	**2800.15**	**p = 0**	**0.92**	**-**	**-**
**NORTHERN CHINA**	**2**	**1013**	**1**	**0.0009[0.0000-0.0037]**	**0.36**	**p = 0.5488**	**0.0**	**0.0101**	**-0.5563 (-0.9800 to -0.1327)**
**EASTERN CHINA**	**2**	**452**	**21**	**0.0460[0.028-0.0672]**	**0.94**	**p = 0.3312**	**0.0**	**0.0635**	**-0.4378 (-0.9003to 0.0246）**
**CENTRAL CHINA**	**2**	**147**	**19**	**0.1293[0.0801-0.1881]**	**0.00**	**p < 0.0001**	**--**	**0.4862**	**-0.2053 (-0.7832 to 0.3726)**
**NORTHWESTERN CHINA**	**12**	**6878**	**723**	**0.1554 [0.0574; 0.2899]**	**615.03**	**p = 0.3376**	**98.2**	**0.1475**	**-0.1679(-0.3951 to 0.0593)**
**SOUTHWESTERN CHINA**	**2**	**373**	**73**	**0.1953 [0.1567; 0.2371]**	**0.92**	**p = 0**	**0.0**	**0.5465**	**-0.1306 (-0.5551 to 0.2939)**
SAMPLING YEAR									
**2016-2025**	3	2783	181	0.3083 [0.0021-0.8192]	141.34	p < 0.0001	98.6	-	-
**2006-2015**	9	3425	1197	0.2990 [0.1346-0.4960]	1694.59	p = 0	98.6	0.9816	-0.0051 (-0.4394 to 0.4292)
**1996-2005**	10	6266	576	0.1445 [0.0381-0.3034]	871.28	p < 0.0001	99.0	0.3723	-0.1941 (-0.6206 to 0.2324)
GENOSPECIES									
B.garinii + B.afzelii	10	6378	1435	0.3264 [0.1401-0.5471]	3151.25	p = 0	99.7	-	-
B.garinii	7	5541	390	0.1441 [0.0319-0.3180]	332.12	p < 0.0001	98.2	0.1854	-0.2173 (-0.5389 to 0.1043)
B.afzelii	1	293	20	0.0683 [0.0423; 0.0999]	0.00	p = 0	-	0.3245	-0.3433 (-1.0261 to 0.3396)
TICK SPECIES									
IXODES HYATTI	1	1	0	0.0000 [0.0000-0.6897]	0.00	-	-	0.3343	-0.6323 (-1.9160 to 0.6514)
IXODES OVATUS	1	35	0	0.0000 [0.0000; 0.0272]	0.00	-	-	0.1427	-0.6323 (-1.4778 to 0.2132)
IXODES PERSULCATUS	22	11151	1791	0.2030 [0.1089; 0.3174]	3960.97	P = 0	99.5	0.5386	-0.1643 (-0.6881 to 0.3594)

IXODES ACUTITARSUS	2	47	33	0.3026 [0.0000-1.0000]	30.93	p < 0.0001	96.8	-	-
IXODES VESPERTILIONIS	1	4	0	0.0000 [0.0000-0.2215]	0.00	P = 0	-	0.1982	-0.6323 (-1.5954 to 0.3308)

NOTE: CI - CONFIDENCE INTERVAL, X2 - CHI-SQUARED. THE SAMPLE-SIZE THRESHOLD OF ⩾30 WAS APPLIED AT THE ORIGINAL STUDY OR INDEPENDENT DATASET LEVEL. SOME SUBGROUP STRATA WERE DERIVED FROM ELIGIBLE STUDIES AND MAY HAVE SMALLER DENOMINATORS; THESE SPARSE STRATA ARE PRESENTED FOR DESCRIPTIVE TRANSPARENCY ONLY AND SHOULD NOT BE INTERPRETED AS INDEPENDENT ESTIMATES OF VECTOR IMPORTANCE OR SPECIES-SPECIFIC RISK.
